# Phytoalexin Phenalenone Derivatives Inactivate Mosquito Larvae and Root-knot Nematode as Type-II Photosensitizer

**DOI:** 10.1038/srep42058

**Published:** 2017-02-07

**Authors:** Runjiang Song, Yian Feng, Donghui Wang, Zhiping Xu, Zhong Li, Xusheng Shao

**Affiliations:** 1 Shanghai Key Laboratory of Chemical Biology, School of Pharmacy, East China University of Science and Technology, Shanghai, 200237, China; 2 College of Life Sciences, Peking University, Beijing 100871, China

## Abstract

Phytoalexins phenalenones (PNs) are phytochemicals biosynthesized inside the plant in responsive to exterior threat. PNs are excellent type-II photosensitizers, which efficiently produce singlet oxygen upon light irradiation. Based on the core functional structure of PNs, novel PN derivatives were synthesized here and their singlet oxygen generating abilities and their phototoxicity were evaluated. At the presence of light, these PNs have photoinduced toxicity towards *Aedes albopictus* larvae and nematode *Meloidogyne incognita*, while the activity lost in the dark. The obvious tissue damage was observed on the treated mosquito larvae and nematode due to the generation of singlet oxygen. Our results revealed the potential of phenalenones as photoactivated agents for mosquito and root-knot nematode management together with light.

Phytochemicals have potential group arrays and novel scaffold architectures for the successful development of biologically functional molecules^1^,^2^,^3^,^4^. Many notable agents with pest management ability are inspired from the phytochemicals, such as neonicotinoids from nicotine^5^, pyrethroids from pyrethrum^6^, carbamates from physostigmine^7^ and 2,4-D herbicide from indoleacetic acid^8^. Therefore, the functional exploration on the phytochemicals will facilitate the understanding of chemical interaction with biological systems and the discovery of safer and new biologically useful compounds.

Phytoalexins are a kind of protecting phytochemicals biosynthesized inside plant in responsive to exterior threat, such as pathogen infections or mechanical injury^9^. They are chemically diverse phytochemicals with varying activities upon the plant species. Phytoalexins have attracted great attentions of scientists from various fields, in particular the agrochemists who are searching for new potential chemicals for pest regulation.

Phototoxic phytoalexins sometimes were used by plant together with the light as a quick defense at the moment of stress. These plant phototoxins are photosensitizers that can generate reactive oxygen species upon absorption of light energy^10^,^11^. A number of plants including Musaceae, Strelitziaceae, Pontederiaceae and Haemodoraceae produce phototoxins containing the phenalenone (PN) structural component^10^,^12^,^13^,^14^. PNs are often found in plants infected or attacked by microorganisms and fungi for killing the invaders. These natural PNs or their modified structures exhibited antifungal^10^,^15^, antiplasmodial^16^ and antiprotozoal activity^17^ and radical scavenging capacity^18^. PN derivatives have also been developed as dental drugs for photodynamic inactivation of oral key bacteria^19^. A recent research by Dirk Hölscher *et al*. revealed that some PN analogues are the main defensive phytoalexins in banana plants attacked by nematode^20^. Since PN is a good photosensitizer with almost 100% quantum yields^10^, the mechanism of defense triggered by phytochemicals PN arouses exceptional interest in the field of prospective applications. We studied herein the ability of PNs to inactivate disease vector mosquito larvae and plant-parasitic nematodes through mechanism involving singlet oxygen production upon light stimuli.

## Results and Discussion

### Molecular design

PN derivatives are the most abundant active phytoalexins isolated from the affected Musaceae^14^. They are excellent photosensitizers that can generate singlet oxygen. We intend to develop the new PN analogues and studied their photoinduced toxicity to the harmful insects. The substituents on the core PN have much influence on the activity level. 9-phenyl-phenalenones (PPN) or 2-hydroxyl-9-phenyl-phenalenones (HPPN) have the rapid and long-lasting antinematode activity to burrowing nematode *Radopholus similis*^20^. Teresa Abad-Grillo *er al.* reported some 9-heterocyclic substituted phenalenone analogues with good antiplasmodial and antiprotozoal activity against *Leishmania amazonensis, Trypanosoma cruzi* and *Plasmodium falciparum*. The above studies indicate the importance of the substituents at 9-position^17^. Therefore, we firstly prepared the 9-substituted PNs analogues (PPNs) to study its photosensitized ability. With a view to utilize these photosensitizer in aqueous media to control mosquito larvae and root-knot nematode, the water soluble derivatives 2-pyridiniium-9-phenyl-phenalenone (PPPN) were synthesized by attaching a pyridinium salt fragment. Meanwhile, the atom fluorine was introduced into the structure due to its particular role in the bioactivity, such as high electronegativity, good hydrophobicity and metabolic stability^21^.

### Phenalenones Synthesis

**Figure 1:**
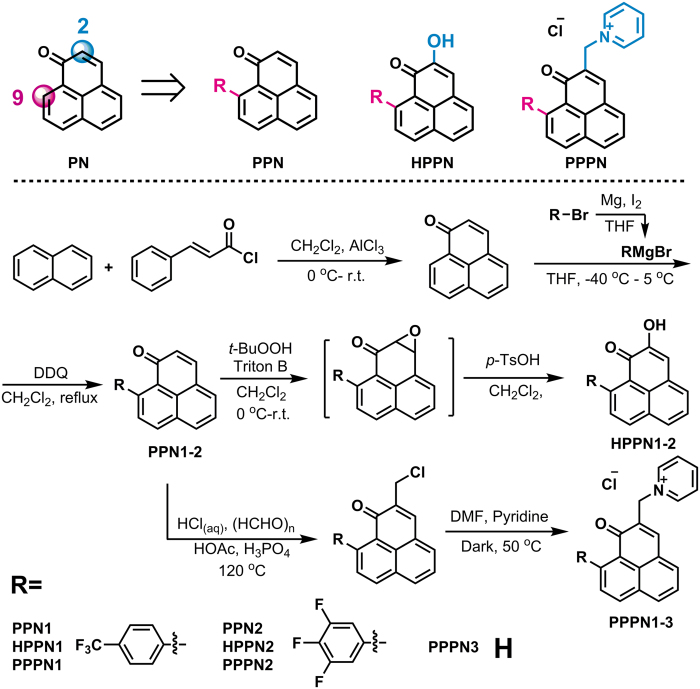
Molecular design of phenalenone derivatives and their synthesis routes.

The synthetic sequence for the required compounds are depicted in Fig. 1. The PN was synthesized according to the reported procedure by reaction of naphthalene and cinnamoyl chloride via Friedel-Crafts reaction and aromatization through elimination of benzene^22^. PPNs were prepared by slow adding of Grignard reagents into PN in tetrahydrofuran at −40 °C and the followed refluxing in dichloromethane catalyzed by 2,3-dichloro-5,6-dicyano-1,4-benzoquinone (DDQ). Epoxidation of PPNs and acid treatment of the intermediary epoxide facilitate the appending of 2-hydroxyl group generating HPPNs^18^. Finally, the pyridinium salt PPPNs was attached through Blanc chloromethylation of PPNs followed by reactions with pyridine^19^.

### Detection of singlet oxygen

**Figure 2:**
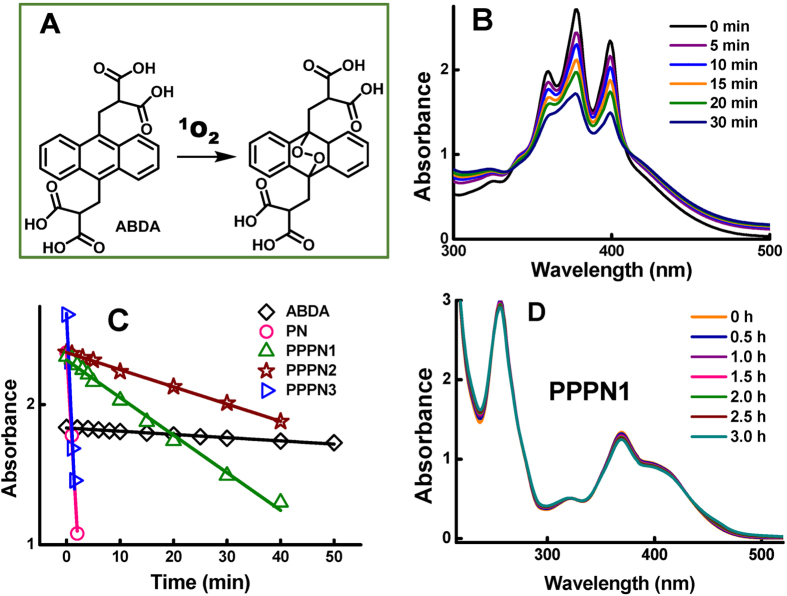
Evaluation on singlet oxygen generating ability. (**A**) Chemical reaction of ABDA with singlet oxygen. (**B**) UV-Vis absorbance spectra of ABDA upon irradiation in the presence of PPPN1. (**C**) Absorbance changes of ABDA at wavelength of 400 nm under irradiation over different periods of time; (**D**) Photostability of PPPN1 upon irradiation of blue light (420 nm).

The ^1^O_2_ photogenerating process and ability of the synthesized compounds were determined through a chemical method based on ^1^O_2_ sensor 9, 10-anthracenediylbis(methylene)dimalonic acid (ABDA)^23^. ABDA rapidly and quantitatively converts to a steady-state endoperoxide product by reacting with singlet oxygen in the presence of photosensitizer (Fig. 2A). Reaction process was monitored by the decrease of absorbance intensity of ABDA upon increasing irradiation at 420 nm (Fig. 2B). The quantum yields of photosensitized ^1^O_2_ generation were calculated from the comparison of quantum yield of PN (Φ_△_ = 1)^24^. PN is an efficient singlet oxygen photosensitizer with ca. 100% quantum yield in a large variety of solvents. It has good photostability under the irradiation wavelength of light and low ability to deactivate singlet oxygen. The water solubility of PPN1-2 and HPPN1-2 was too poor to measure their quantum yields. The quantum yields of PPPN1 and PPPN2 are measured to be 0.12 and 0.09 in acetonitrile, respectively, indicating that 9-phenyl installation reduced the efficiency of ^1^O_2_ generation. This decrease is caused by the intramolecular charge-transfer from the phenyl to the electron-deficient PN fragment^10^. Introduction of hydroxyl group at 2-position of PPN led to the further decrease of quantum yields with values of about 3 orders of magnitude lower than that of parent PN. The quantum yields of PPPN1 and PPPN2 were about 26-fold and 31-fold lower than that of PN, respectively. This trend could also be reflected in the time-dependent absorbance change of ABDA, in which PN or PPPN3 induced much faster extinction of ABDA (Fig. 2C). Although the ^1^O_2_ generating efficiency of PPPN1 and PPPN2 is low, the relatively-slow generation of singlet oxygen has its value for practical application in plants because the balance between defensing outside threaten and autotoxicity should be taken into accounts^10^. The PPPNs is resistance to photobleaching, since no significant photo degradation of PPPN1 was detected after 3 h exposition to light (Fig. 2D).

### Photoinduced activity to mosquito larvae

**Figure 3:**
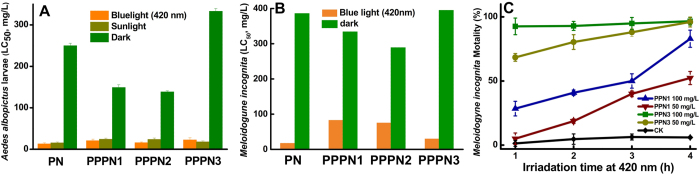
Photoactivated activity evaluation. (**A**) The activity of phenalenones with or without irradiation against *Aedes* albopictus larvae. (**B**) Mortality of phenalenones against *Meloidogune incognita* upon irradiation of blue light (420 nm) at 50 mg/L. (**C**) Mortality of phenalenones against *Meloidogune incognita* under different irradiation time.

Mosquitos are vectors of some of humanity’s deadly diseases causing one millions deaths worldwide annually and almost everyone has suffered from their bite allergy^25^. This aquatic and transparent insect is the potential target for action of photosensitizer. The intrinsic and light-dependent toxicity of these PN derivatives were evaluated against *Aedes albopictus* larvae and the results were summarized in Table 1. Taking the water solubility and ^1^O_2_ producing ability into account, the PPPN1-3 together with PN were tested for photoactivated activity. The light sources used for ^1^O_2_ production are blue-light (420 nm) or sunlight, although the maximum absorbance wavelength of PN derivatives are around 370 nm. The reason for such selection is to avoid the harmful irradiation of UV-light that might cause damage to insects. Blue light (420 nm) alone do not have any detrimental effects to the larvae. PN derivatives in the absence of light showed very low killing potency to mosquito larvae with large LC_50_ values ( >140 mg/L). A trend emerged that the assembling of phenyl component at 9-position led to a 2-fold activity decrease (Fig. 3A, PPPN1 and PPPN2 vs. PPPN3). When exposed to blue light, a significant activity enhancement for all the tested compounds was observed with LC_50_ ranging from 13.0–22.5 mg/L, indicating 7–19 folds activity increase. This phenomenon that PN derivatives needs work together with oxygen and light to exhibit their efficacy demonstrated a photosensitized mechanism. To our delight, the same activated effects were achieved through sunlight irradiation, implying a clean, energy-saving and easily available light source in practical application. The slight decrease of activity of pyridinium form PPPN1-3 in comparison with PN might in part attribute to their high water solubility which limit effective tissue distribution of the chemical^11^,^26^,^27^.

**Figure 4:**
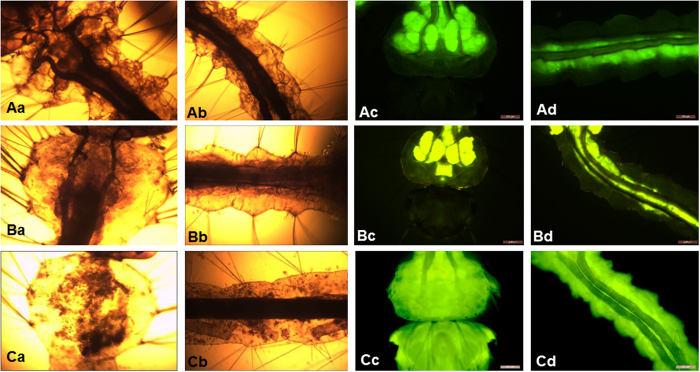
Microscopy (**Aa,b,Ba,b,Ca,b**) and fluorescence (**Ac,d,Bc,d, Cc,d**) images of Aedes albopictus larvae. The larvae was treated by PPPN1 with or without irradiation at wavelength of 420 nm. Fluorescence dye FITC (100 mg/L) was used to counterstain to visualize the *Aedes* larvae. Scale bar, 200 *μ*m for fluorescence images and 100 *μ*m for microscopy images. Aa–Ad: head and body images of *Aedes* larvae irradiated by blue light (420 nm); Ba–Bd: head and body images of *Aedes* larvae treated by PPPN1 under dark; Ca–Cd: head and body images of *Aedes* larvae treated by PPPN1 and irradiated by blue light (420 nm).

**Figure 5:**
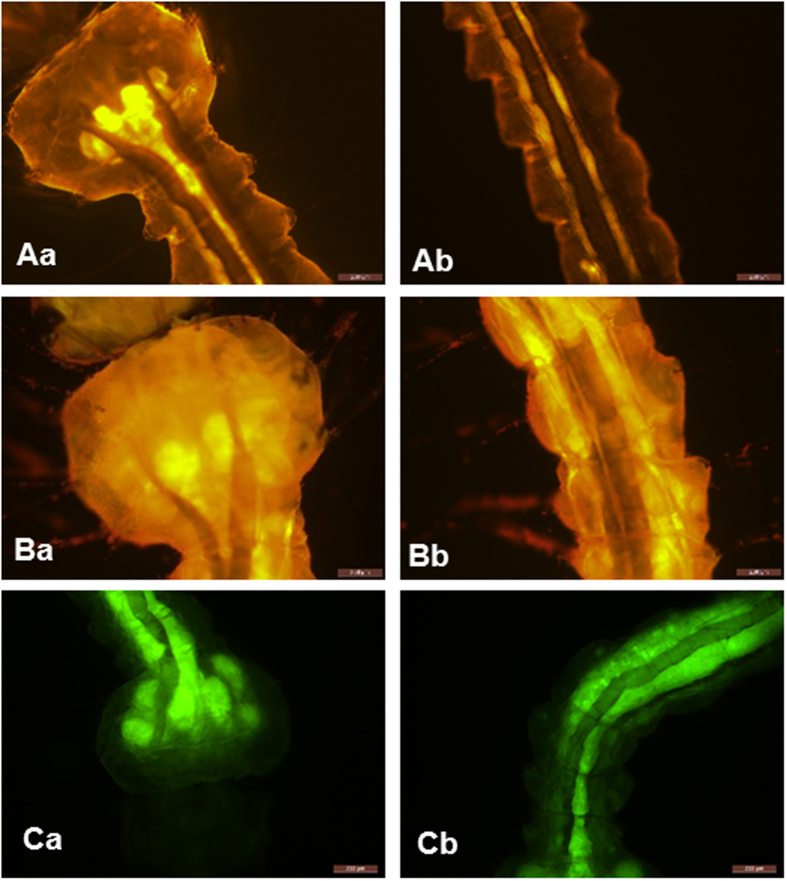
Fluorescence images of impaired *Aedes albopictus* larvae. Fluorescence dye FITC (100 mg/L) was used to counterstain to visualize the *Aedes* larvae. Aa-Ab: head and body images of *Aedes* larvae treated by PPPN1 (100 mg/L) under dark; Ba–Bb: head and body images of *Aedes* larvae treated by PPPN1 (100 mg/L) and irradiated by blue light (420 nm); Ca–Cb: head and body images of *Aedes* larvae treated by fipronil (10 mg/L).

Biological dysfunction induced by ^1^O_2_ includes cell death, membrane damage and enzyme inactivation^11^. In order to understand the damage occurring to the larvae, the microscopy and fluorescence images of mosquito larvae were acquired (Fig. 4). The pictures were collected 24 h after application of PPPN1. The blue light (420 nm) was irradiated in the first four hour of incubation. No significant adverse effect after exposure to blue light was detected in the control treatment without photosensitizer (Fig. 4, Aa and Ab) and the fluorescein isothiocyanate (FITC) concentrated mainly in the digestive system (Fig. 4Ac and Ad), that is, gastric caeca and guts lumen^26^,^28^. Incubation with PPPN1 but without light (dark control) can not cause any damage to the larvae (Fig. 4Ba,b,c and d), proving the inactivity of the compound in the dark. *Aedes* larvae treated with PPPN1 plus blue light underwent obvious tissue damage and rupture (Fig. 4a and b) accompanied with the fluorescence dye leaking to the whole body (Fig. 4Cc and d). As PN has weak intrinsic yellow fluorescence, it was therefore used as both photosensitizer and staining agent for visualization study (Fig. 5Aa,b,a and b) to elucidate the partitioning of the photosensitizer among the different organs of mosquito larvae. When PN was administrated without irradiation, the PN mainly accumulated in the digestive system (Fig. 5Aa and b) and afterward blue light stimuli made it penetrate into other body parts (Fig. 5Ba and b). The above observed symptoms is different to that of larvaes poisoned by fipronil and stained by FITC (Fig. 5Ca and b). This observation demonstrated that initial allocation of photosensitizer around gut would destroy gut cells and the subsequent photosensitizer diffusion caused further photo damage to the larvae, leading to final death of the insect.

### Photoinduced activity to root-knot nematode

Plant parasitic nematodes cause great crop losses annually. In the nematode-resistant banana cultivar, the main isolated defensive phytoalexins were phenylphenalenones which have the nematostatic and nematicidal activity towards burrowing nematode *Radopholus similis*. Due to the potential photo toxicity of photosensitizer to nematodes^29^, the biological responses of phenalenones PN and PPPN1-3 were evaluated here towards root-knot nematode *Meloidogyne incognita*. All the tested compounds have low dark activity. Similar synergistic effects of light to the synthesized PN derivatives were observed with the increase in the nematicidal activity (Fig. 3B and Table 2). Although nematode was sensitive to UV light^29^, no obvious photobiological responses were detected under the wavelength of light used here. The increased sensitivity of nematode correlated closely with the increased length of light exposure^30^. Complete photocidal effects could be achieved by 1 h light stimuli for PPPN3 at concentration of 100 mg/L, while the photo toxicity of low dosage PNs increased gradually with prolongation of irradiation (Fig. 3C).

**Figure 6:**
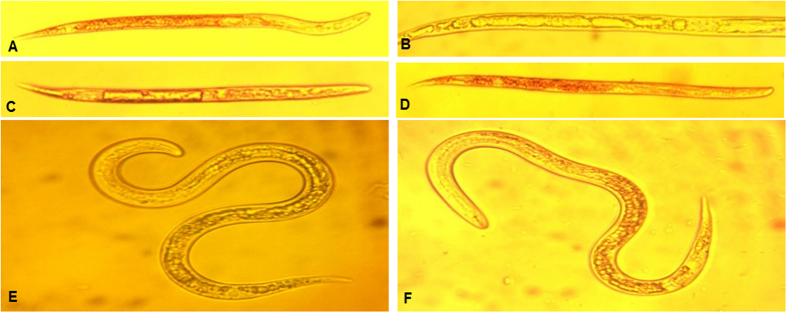
Microscopy images of *Meloidogune incognita*. (**A**) *Meloidogune* treated by PPPN1 (100 mg/L) under dark; (**B**) *Meloidogune* treated by PPPN1 (100 mg/L) and irradiated by blue light (420 nm); (**C**) *Meloidogune* treated by PPPN3 (100 mg/L) and irradiated by blue light (420 nm); (**D**) *Meloidogune* treated by avermectin (5 mg/L) under dark; (**E**) Untreated *Meloidogune* under dark. (**F**) *Meloidogune* irradiated by blue light (420 nm).

Nematode microscopy images of PPPNs-treated nematodes exerted obvious morphological abnormalities (Fig. 6B and C) and larger differences with the dark control (Fig. 6A) and blank control (Fig. 6E and F, with or without irradiation, respectively). Cell destroy and merging were observed in the body of PPPN-loaded larvae after imposition of light, presenting a droplet-like appearance. Sole treatment of nematode with PPPN1 did not lead to the formation of such large droplets which is opposed to the previous observation that yellow droplets appeared after application of anigorufone in *Radopholus similis*^20^. From present observation, the formation of droplets might be partly attributed to the photosensitization, since no dark controls were conducted in anigorufone-treated *R. similis*^20^. There is also a possibility that poorly water-soluble anigorufone mainly accumulated in the lipid droplets which prevents the efficient photosensitization process^31^. The water soluble PPPNs used in our study are more efficient for the generation of ^1^O_2_ upon light irradiation, which led to destroy and merge of the tissue components generating a droplet-like shape. In contrast, the nematode poisoned by avermectin did not form such droplets, indicating the unique toxic features caused by singlet oxygen.

The level of the photoinduced activity is preferentially associated with the ^1^O_2_ generating ability, light intensity and distribution in the body^11^,^32^. The distribution is determined by hydrophobicity, moreover, it also relates closely with the type of the target insects^11^. Cell membranes are preferential location for hydrophobic photosensitizer, while passive and active diffusion processes are involved in the hydrophilic one^32^. Amphiphilic photosensitizer is particular efficient in crossing the lipid cell membrane and subsequent diffusing in water-based environment inside the cell^11^. Previously studies showed that the primary oxidative modification sites were the membranes of midgut wall^11^. Despite of large difference in the ^1^O_2_ generating efficiency, PN and PPPNs have the same level of photo killing activity. The low water solubility of PN limits its efficient ingestion by the insect, although it has high quantum yields. In case of PPPNs, amphiphilic feature make them easily arrive at the target site, offsetting the weakness of relatively low ^1^O_2_ generating ability. Another possible explanation for the uncorrelation of the quantum yields with the activity is the limited localized oxygen concentration inside the insect. The generation ability of singlet oxygen correlates closely with the localized oxygen concentration. Inside the body, the localized oxygen was consumed up quickly upon irradiation, which prevents the further generation of the singlet oxygen.

## Conclusion

We described here the ability of phenalenones derivatives to inactivate the mosquito larvae and root-knot nematode through generation of singlet oxygen upon light irradiation. The utilization of photochemical mechanism for controlling harmful species processes several substantial advantages including dark inactiveness, no cross-resistance and controllable activity. The present study provided a basic understanding of PN derivatives as potential mosquito- or nematode- management chemicals. For practical pest control, novel application methods are needed to be developed to efficiently use them, such as immobilization of the photosensitizer to recover or reuse them, anchored in the wall paintings, combining with the bait and coated on the surface of small beads. Switching the absorption band to the red-light range through structural modification is another challenge for better penetration to biological tissues and efficient use of sunlight, since using sustainable natural light is the growing trend in the future. Endeavors in this direction may lead to the development of improved strategies for controlling mosquito-borne diseases or root-knot nematodes.

## Methods

### Synthesis PN derivatives

The instruments, chemicals, general synthetic procedures and the structural characterization of PNs were provided in supplementary files.

### Chemical oxidation detection of singlet oxygen

An ABDA-based oxidation method was used to assess the capability of PNs to generate ^1^O_2_^23^. A mixture of PN derivative (0.3 mmol in 3 mL in ultrapure water) and 50 *μ*L ABDA solution (10 mmol/L in DMSO) was irradiated with a light-emitting diode lamp (blue light, 420 nm, 7 W). The change of ABDA absorption at 400 nm was collected using Varian Cary 100 UV-vis spectrophotometer as a function of irradiation time. The control experiment was conducted using 3 mL ultrapure water containing above-mentioned 50 *μ*L ABDA solution upon the same irradiation but in the absence of PN derivative. The ^1^O_2_ quantum yield was calculated according to Equation (1) using PN as reference (Φ_PN_ = 1), where Φ is the quantum yield of ^1^O_2_ and K is the slope of the bleaching curve, R denotes reference, and S the sample.


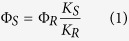


### Photoactivated activity of *Aedes albopictus* larvae

Photoactivated larvicidal activity was determined using previously-described methods^33^. The tested insects were the 4th-instar larvae of *A. albopictus* which were obtained from National South Pesticide Innovation Center in Shanghai, China. PN was dissolved and serially diluted with acetone and PPPN1-3 was dissolved and serially diluted with dechlorinated water. Each serial solution (0.2 mL) was added to a beaker containing 10 mL of dechlorinated water, and then 15 larvae were transferred into the dactylethrae. Two sets of experiments were performed for each compounds, one of which was for light-treated trials, and another was cultivated in the dark throughout the trials. The light-treated groups were irradiated with blue light for 3 h after 3 h incubation in dark, and then returned to darkness for 24 h incubation. The light intensity of the testing solution surface is about 50 W/m^2^. The average mortality of three replications at each concentration was calculated, and the LC_50_ value was determined. All the experiments were conducted at least three times with three replicates in each case. The microscopy images of *A. albopictus* larvae were taken by a polarizing microscope.

### Fluorescent staining of *Aedes albopictus* larvae

Fluorescein isothiocyanate (FITC) uptake was analyzed by soaking *A. albopictus* larvaes in 10 mL of 100 mg/L FITC in M9 buffer (43.6 mM Na_2_HPO_4_, 22 mM KH_2_PO_4_, 2.1 mM NaCl, 4.7 mM NH_4_Cl) with 100 mg/L PPPN1, 10 mg/L fipronil or without any other compound. Three sets of experiments were performed for each compound, one of which was for light-treated trials, and another was cultivated in the dark throughout the trials. After 24 h, the larvaes were washed three times with M9 buffer. FITC uptake was observed by fluorescence, using a fluorescence microscope with blue exciting light.

### Photoactivated activity against root-knot nematode

*M. incognita* population were grown on *Lycopersicon esculentum* plants in a greenhouse, and collected as described by Rosso and associates^34^. Then hatched **J2** were collected as described in Petri Plate Technique^35^. PN and PPPN**1**–**2** were dissolved with DMF and serially diluted with dechlorinated water. Each serial solution (0.05 mL) was added to three wells of 96-well platers and then 0.05 mL water containing root-knot nematode (J2) was transferred into the 96-well plate. Three sets of experiments were performed for each compound, one of which was for light-treated trials, and another was cultivated in the dark throughout the trials. The light intensity of the testing solution surface is about 50 W/m^2^. After 3 h incubation in dark, the light-treated groups were irradiated with blue light for 1 h, 2 h, 3 h and 4 h, and then returned to darkness for 24 h incubation. The average mortality of three replications at each concentration was calculated. All the experiments were conducted at least three times with three replicates in each case. The microscopy of *M. incognita* were taken by a polarizing microscope.

## Additional Information

**How to cite this article**: Song, R. *et al*. Phytoalexin Phenalenone Derivatives Inactivate Mosquito Larvae and Root-knot Nematode as Type-II Photosensitizer. *Sci. Rep.* **7**, 42058; doi: 10.1038/srep42058 (2017).

**Publisher's note:** Springer Nature remains neutral with regard to jurisdictional claims in published maps and institutional affiliations.

## Supplementary Material

Supplementary Information

## Figures and Tables

**Table 1:** The activity of phenalenones with or without irradiation against *Aedes albopictus* larvae.

Compound	LC_50_ (mg/L)			Ratio (dark/blue light)	Ratio (dark/sunlight)
	Blue light (420 nm)	Sunlight	Dark		
PN	13.0 ± 2.5	15.4 ± 2.1	250 ± 5.9	19.2	16.2
PPPN1	15.8 ± 1.9	23.8 ± 3.1	140 ± 2.6	8.8	5.8
PPPN2	20.7 ± 3.7	24.1 ± 2.7	149 ± 6.8	7.2	6.2
PPPN3	22.5 ± 5.3	17.9 ± 2.3	333 ± 6.4	14.8	18.6

**Table 2:** The activity of phenalenones with or without irradiation against *Meloidogune incognita*.

Compound	LC50 (mg/L)		Ratio (dark/blue light)
	Blue light (420 nm)	Dark	
PN	17.3 ± 1.7	386 ± 6.4	22.1
PPPN1	83.7 ± 3.8	334 ± 7.5	4.0
PPPN2	76.2 ± 4.1	289 ± 5.6	3.8
PPPN3	30.9 ± 2.6	395 ± 7.0	16.6
