# Supplementary material for: Phytoalexin Phenalenone Derivatives Inactivate Mosquito Larvae and Root-knot Nematode as Type-II Photosensitizer
Source: Sci Rep. 2017 Feb 7;7:42058. doi: 10.1038/srep42058 (PMC5294642; doi:10.1038/srep42058)
Supplement: Supplementary Information [file srep42058-s1.pdf]

Supplementary Information

Phenalenone Phytoalexin Derivatives Inactivate Mosquito Larvae and Root-knot Nematode as Type-II Photosensitizer

Runjiang Song[a,‡], Yian Feng[a,‡], Donghui Wang[b], Zhiping Xu[a], Xusheng Shao[a,*], Zhong Li[a]

[a] *Shanghai Key Laboratory of Chemical Biology, School of Pharmacy, East China University of Science and Technology, Shanghai, 200237, China.* [b] *College of Life Sciences, Peking University, Beijing 100871, China.*

Correspondence and request for materials should be addressed to X.S. (shaoxusheng@ecust.edu.cn, +86-21-64253967)

Table of Contents

1. Quantum yields for singlet oxygen generation 2
1. General information 2
2. Synthesis of the compounds 2
   2.1 Synthesis of PPN 2
   2.2 General procedure for the synthesis of PPN 3
   2.3 General procedure for the synthesis of HPPN 3
   2.4 General procedure for the synthesis of PPPN 3
3. Characterization data of products 4
4. Copies of ^{1}H and ^{13}C NMR 6
5. Chemical oxidation detection of singlet oxygen 16
6. Photoactivated insecticidal activity 17
   6.1 Photoactivated activity of *Aedes albopictus* larvae 17
   6.2 FITC uptake 17
   6.3 Photoactivated activity of root-knot nematode 17
References 18

1. Quantum yields for singlet oxygen generation

Table S1. Quantum yields for singlet oxygen generating of phenalenones.

Compound	Quantum yield ($^1O_2$, Φ_Δ)	
	Water	Acetonitrile
PN	1	1
PPN1	-----	0.12
PPN2	-----	0.09
HPPN1	-----	0.02
HPPN2	-----	0.03
HPPN3	-----	0.07
PPPN1	0.038	0.18
PPPN2	0.032	0.16
PPPN3	1.23	-----

1. General information

^1H NMR and ^{13}C NMR spectra were recorded on BrukerAM-400 (^1H at 400 MHz, ^{13}C at 100 MHz) spectrometer with $CDCl_3$, D_2O or DMSO-d_6 as the solvent and TMS as the internal standard. Chemical shifts are reported in δ (parts per million) values. High resolution electron mass spectra (ESI-TOF) were performed on a Micromass LC-TOF spectrometer. Analytical thin-layer chromatography (TLC) was carried out on precoated plates (silica gel 60 F254), and spots were visualized with ultraviolet (UV) light. The following abbreviations were used to explain the multiplicities: s = singlet, d = doublet, t =triplet, q = quartet, m = multiplet, coupling constant (Hz) and integration. The pictures were taken by a polarizing microscope (Jiangnan, XPL-2) or a fluorescence microscope (Leica, DMI30000B).

2. Synthesis of the compounds

2.1 Synthesis of PPN

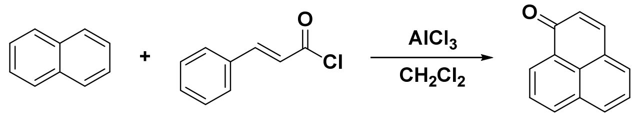

Alchlor (30 mmol, 4.00 g) and naphthalene (10 mmol, 1.28 g) were added to 10 mL CH_2Cl_2, the mixture was cooled to room temperature. Cinnamoyl chloride (10 mmol, 1.67 g) was added in succession under 0 °C, and the mixture was taken to reflux for 3 h. Then the reaction mixture was cooled with iced hydrochloric acid and filtered. The filtrate was extracted with methylene chloride. The solids were repeatedly boiled with hot methylene chloride and filtered until the filtrate became colorless. All the organic extracts were combined, dried over anhydrous sodium sulfate, and taken down on a rotary evaporator to give a yellow solid. The crude product was further purified by column chromatography. Yield 68%.[1]

2.2 General procedure for the synthesis of PPN

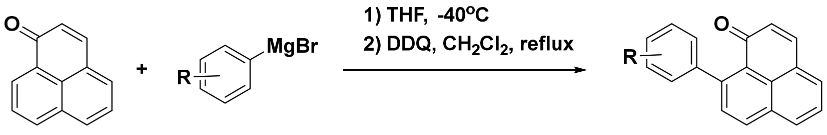

PN PPN

PN (0.004 mol, 0.72 g) was dissolved in THF (10 mL). The solution was added dropwise to the appropriate Grignard reagent (0.006 mol, 1 M in THF, prepared from respective bromide and Mg) via a syringe under -40 °C. After stirring for 20 min, the reaction was moved to ice-bath and quenched with an aqueous saturated solution of NH_4Cl. The aqueous phase was extracted with CH_2Cl_2 and the combined organic layer was washed with brine, dried over Na_2SO_4. The solvent was evaporated and the residue was dissolved in CH_2Cl_2 and treated with DDQ (0.004 mol, 0.908 g). The reaction was refluxed for 3 h, filtered to move the precipitate. The crude product was further purified by column chromatography.[2]

2.3 General procedure for the synthesis of HPPN

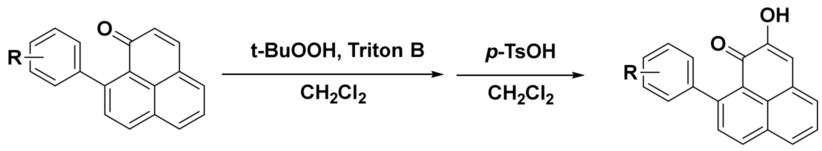

PPN HPPN

PPN (0.002 mol) was dissolved in dichloromethane and cooled at 0 °C, then treated with Triton B (0.003 mmol, CH_2Cl_2) and t-butylhydroperoxide (0.003 mmol) for 5h. The reaction was quenched with NH_4Cl, the organic phase was separated, dried and evaporated. The residue was dissolved in dichloromethane and *p*-toluenesulfonic acid (0.002 mmol) was added. The reaction was stirred until complete as monitored by TLC. The final product was purified by column chromatography, affording deep-yellow solids.[3]

2.4 General procedure for the synthesis of PPPN

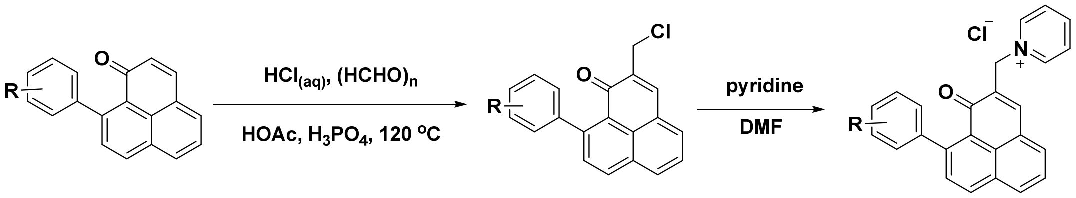

PPN ClPPN PPPN

PPN (0.002 mol) and paraformaldehyde (0.005 mol, 0.15 g) in 8 mL of acetic acid and 5 mL of phosphoric acid (85%) was heated to 120 °C. Hydrochloric acid (36% w/w, 4 mL) was added over a period of 10 min. The brown solution was heated for further 8 h. After cooling of the solution to room temperature, 20 mL of ice cold distilled water was added. The reaction mixture was carefully neutralized with a saturated solution of K_2CO_3. The product was extracted with dichloromethane (3 × 100 mL). The combined organic layers were dried over Na_2SO_4 and concentrated. Further purification was carried out by column chromatography.

ClPPN (0.001 mol) was dissolved in DMF (2 mL). The pyridine (0.017 mol, 1.4 g) in DMF (2 mL) was added dropwise. The reaction was stirred at room temperature in dark for 24 h, then heated to

50 °C for 3 h. After cooling of the reaction to room temperature, diethyl ether (10 mL) was added to precipitate the product. The precipitate was washed several times with diethyl ether.[4]

3. Characterization data of products

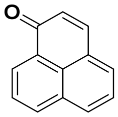

1*H*-phenalen-1-one

Isolated yield: 62.8%. Yellow solid; mp: 150.7~153.8 °C; ^{1}H NMR (400 MHz, $CDCl_3$) δ = 8.64 (dd, *J* = 7.4, 0.8, 1H), 8.21 (d, *J* = 8.0, 1H), 8.03 (d, *J* = 8.3, 1H), 7.78 (m, *J* = 7.80 – 7.75, 3H), 7.63 – 7.57 (m, 1H), 6.74 (d, *J* = 9.8, 1H).

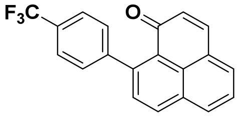

9-(4-(trifluoromethyl)phenyl)-1*H*-phenalen-1-one (PPN1)

Isolated yield: 73.5%. Orange-yellow solid; mp: 191.4~193.5 °C; ^{1}H NMR (400 MHz, $CDCl_3$-*d₁*) *δ* 8.21 (d, *J* = 8.3, 1H), 8.07 (d, *J* = 8.1, 1H), 7.81 (d, *J* = 6.9, 1H), 7.71 (dd, *J* = 8.9, 3.2, 3H), 7.65 (dd, *J* = 8.1, 7.2, 1H), 7.54 (d, *J* = 8.3, 1H), 7.46 (d, *J* = 8.0, 2H), 6.59 (d, *J* = 9.7, 1H) ppm; ^{13}C NMR (100 MHz, $CDCl_3$-d_1) *δ* 185.65, 146.82, 145.84, 140.83, 134.06, 132.08, 131.88, 131.83, 130.92, 130.24, 129.18 (q, J_{CF} = 32.5), 128.51, 128.24, 126.83, 126.09, 125.25 (q, J_{CF} = 3.7), 124.42 (q, J_{CF} = 270.4) ppm; ^{19}F (376 MHz, $CDCl_3$-d_1) *δ* -62.35 (s, 3F) ppm. HRMS (EI): m/z calcd for $C_{20}H_{11}F_3O$ $[M]^+$, 324.0762, found, 324.0753.

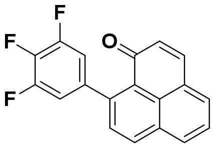

9-(3,4,5-trifluorophenyl)-1*H*-phenalen-1-one (PPN2)

Isolated yield: 67.8%. Yellow solid; mp: 204.7~206.9 °C; ^{1}H NMR (400 MHz, $CDCl_3$-d_1) *δ* 8.20 (d, *J* = 8.3, 1H), 8.06 (d, *J* = 8.2, 1H), 7.82 (d, *J* = 6.9, 1H), 7.72 (d, *J* = 9.7, 1H), 7.69 – 7.63 (m, 1H), 7.51 (d, *J* = 8.3, 1H), 7.00 – 6.90 (m, 2H), 6.60 (d, *J* = 9.7, 1H) ppm; ^{13}C NMR (100 MHz, $CDCl_3$-d_1) *δ* 185.41, 153.57 – 151.87 (m), 150.09 – 149.80 (m), 143.85, 140.88, 139.65 – 138.31 (m), 134.14, 132.21, 131.99, 131.90, 130.62, 130.22, 128.50, 128.17, 127.02, 126.12, 112.27 (dd, J_{CF} = 16.2, 5.7 Hz) ppm. HRMS (EI): m/z calcd for $C_{19}H_9F_3O$ $[M]^+$, 310.0605, found, 310.0604.

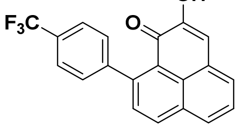

2-hydroxy-9-(4-(trifluoromethyl)phenyl)-1*H*-phenalen-1-one (HPPN1)

Isolated yield: 30.0%. Yellow solid; mp: 223.9~225.3 °C; ^{1}H NMR (400 MHz, $CDCl_3$-d_1) *δ* 8.28 (d, *J* = 8.2, 1H), 7.97 (d, *J* = 8.2, 1H), 7.81 – 7.71 (m, 3H), 7.67 – 7.61 (m, 1H), 7.56 (d, *J* = 8.2, 1H), 7.49 (d, *J* = 8.2, 2H), 7.15 (s, 1H), 6.95 (s, 1H, -OH) ppm; ^{13}C NMR (100 MHz, $CDCl_3$-d_1) *δ* 180.27, 149.69, 147.07, 146.13, 135.89, 131.86, 131.14, 130.54, 129.66 (q, J_{CF} = 32.5), 128.30, 127.42, 125.64, 125.26 (q, J_{CF} = 3.7), 124.90, 124.29 (q, J_{CF} = 270.4), 123.66, 122.94, 113.18 ppm; ^{19}F (376

MHz, $CDCl_3$-*d1*) δ -62.36 (s, 3F) ppm. HRMS (EI): m/z cacld for $C_{20}H_{11}F_3O_2$ $[M]^+$, 340.0711, found, 340.0706.

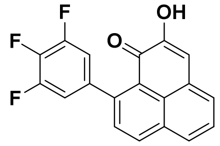

2-hydroxy-9-(3,4,5-trifluorophenyl)-1*H*-phenalen-1-one (HPPN2)

Isolated yield: 34.5%. Orange solid; mp: 201.5~203.3 °C; ^{1}H NMR (400 MHz, $CDCl_3$-*d1*) δ 8.27 (d, *J* = 8.2, 1H), 7.96 (d, *J* = 8.1, 1H), 7.77 (d, *J* = 7.0, 1H), 7.68 – 7.61 (m, 1H), 7.54 (d, *J* = 8.2, 1H), 7.15 (s, 1H), 7.02 – 6.95 (m, 2H), 6.94 (s, 1H, -OH) ppm; ^{13}C NMR (100 MHz, $CDCl_3$-*d1*) δ 180.06, 152.58 – 152.02 (m), 150.14 – 149.70 (m), 149.61, 145.01, 138.18 – 137.95 (m), 135.98, 131.99, 131.31, 130.22, 129.92, 128.86, 127.62, 124.83, 123.72, 113.24, 112.42 (dd, J_{CF} =16.3, 5.9) ppm; ^{19}F (376 MHz, $CDCl_3$-*d1*) δ -134.42 – -134.63 (m, 2F), -161.90 – -162.04 (m, 1F) ppm. HRMS (EI): m/z cacld for $C_{19}H_9F_3O_2$ $[M]^+$, 326.0555, found, 326.0554.

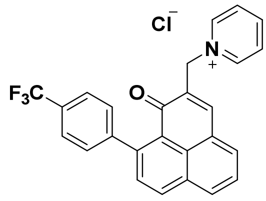

1-((1-oxo-9-(4-(trifluoromethyl)phenyl)-1*H*-phenalen-2-yl)methyl)pyridin chloride (PPPN1)

Isolated yield: 96.2%. Yellow solid; mp: 207.6~209.6 °C; ^{1}H NMR (400 MHz, DMSO-*d6*) δ 9.20 (s, 2H), 8.61 (t, *J* = 7.7, 1H), 8.56 (d, *J* = 8.3, 1H), 8.41 (d, *J* = 7.3, 2H), 8.20 (d, *J* = 7.0, 1H), 8.14 (t, *J* = 6.9, 2H), 7.87 (t, *J* = 7.6, 1H), 7.77 (d, *J* = 8.0, 2H), 7.66 (d, *J* = 8.2, 1H), 7.53 (d, *J* = 7.9, 2H), 5.72 (s, 2H) ppm; ^{13}C NMR (100 MHz, DMSO-*d6*) δ 183.18, 146.76, 145.82, 145.66, 145.19, 143.28, 135.38, 134.38, 133.40, 132.22, 131.54, 131.23, 128.82, 127.87, 127.48 (q, J_{CF} = 31.5), 127.47, 127.33, 126.60, 124.96 – 124.89 (m), 124.77, 124.42 (q, J_{CF} = 270.3), 59.47 ppm; ^{19}F (376 MHz, DMSO-*d6*) δ -60.72 (s, 3F) ppm. HRMS (ESI): m/z cacld for $C_{25}H_{15}F_3NO$ $[M]^+$, 402.1106, found 402.1105.

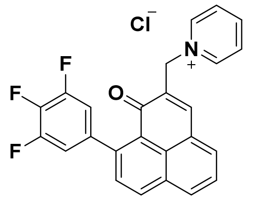

1-((1-oxo-9-(3,4,5-trifluorophenyl)-1*H*-phenalen-2-yl)methyl)pyridin chloride (PPPN2)

Isolated yield: 93.8%. Yellow solid; mp: 145.1~146.8 °C; ^{1}H NMR (400 MHz, DMSO-*d6*) δ 9.20 (d, *J* = 5.5 Hz, 2H), 8.61 (t, *J* = 7.7 Hz, 1H), 8.55 (d, *J* = 8.3 Hz, 1H), 8.43 – 8.34 (m, 2H), 8.20 (d, *J* = 7.0 Hz, 1H), 8.14 (t, *J* = 7.0 Hz, 2H), 7.86 (t, *J* = 7.6 Hz, 1H), 7.69 (d, *J* = 8.3 Hz, 1H), 7.39 – 7.29 (m, 2H), 5.73 (s, 2H) ppm; ^{19}F (376 MHz, DMSO-*d6*) δ -136.30 (dd, *J* = 21.7, 9.0 Hz, 2F), -164.20 (tt, *J* = 21.8, 6.7 Hz, 1F) ppm. HRMS (ESI): m/z cacld for $C_{26}H_{17}F_3NO$ $[M]^+$, 416.1262, found, 416.1261.

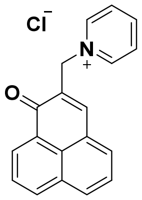

1-((1-oxo-1*H*-phenalen-2-yl)methyl)pyridin chloride (PPPN3)

Isolated yield: 92%. Yellow solid; mp: 156.8~158.1 °C; ^{1}H NMR (400 MHz, D_2O-*d*$_2$) δ 8.82 (d, *J* = 4.5, 2H), 8.47 (t, *J* = 7.0, 1H), 7.99 (s, 2H), 7.55 (s, 1H), 7.38 (t, *J* = 8.0, 2H), 7.28 (d, *J* = 6.2, 1H), 7.20 (d, *J* = 6.8, 1H), 7.09 (d, *J* = 7.1, 1H), 6.83 (t, *J* = 7.1, 1H), 5.19 (s, 2H) ppm; ^{13}C NMR (100 MHz, D_2O-*d*$_2$) δ 183.76, 146.19, 145.19, 144.67, 136.31, 134.85, 134.12, 130.48, 130.29, 129.16, 128.00, 126.84, 126.65, 126.14, 124.99, 124.58, 60.36 ppm. HRMS (ESI): m/z cacld for $C_{19}H_{14}NO$ $[M]^+$, 272.1075, found, 272.1077.

4. Copies of ^{1}H and ^{13}C NMR

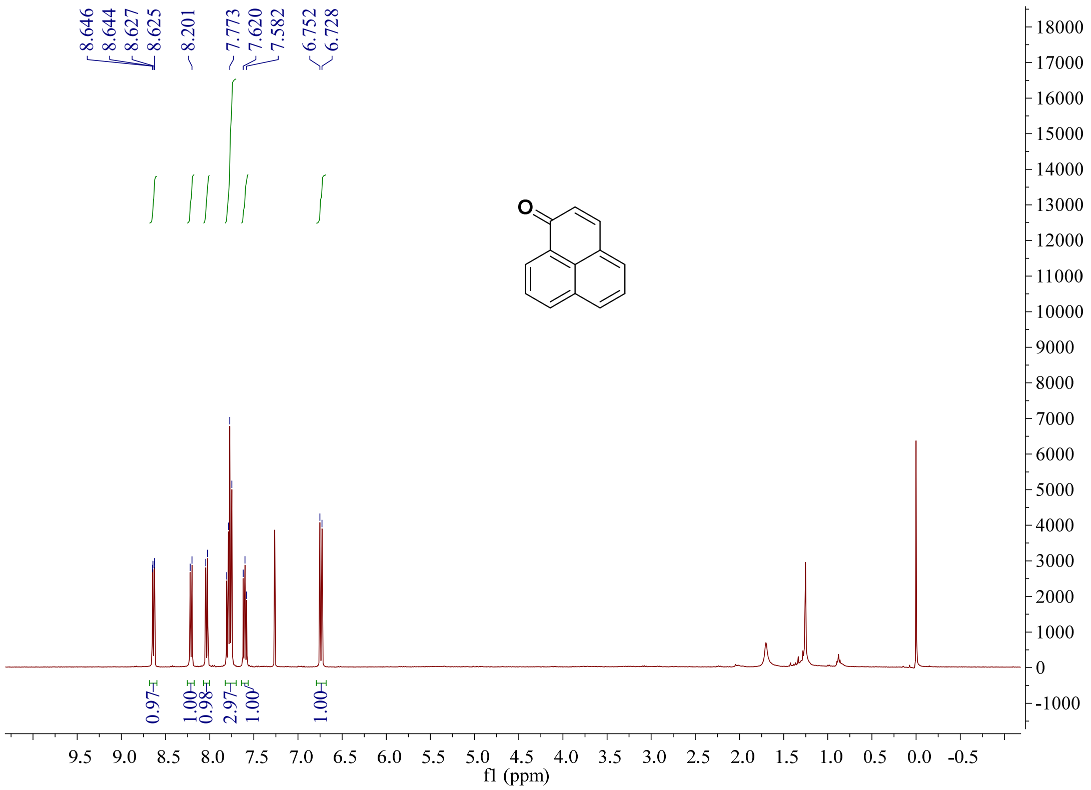

Figure S1. ^{1}H NMR (400 MHz, $CDCl_3$) spectra of PN

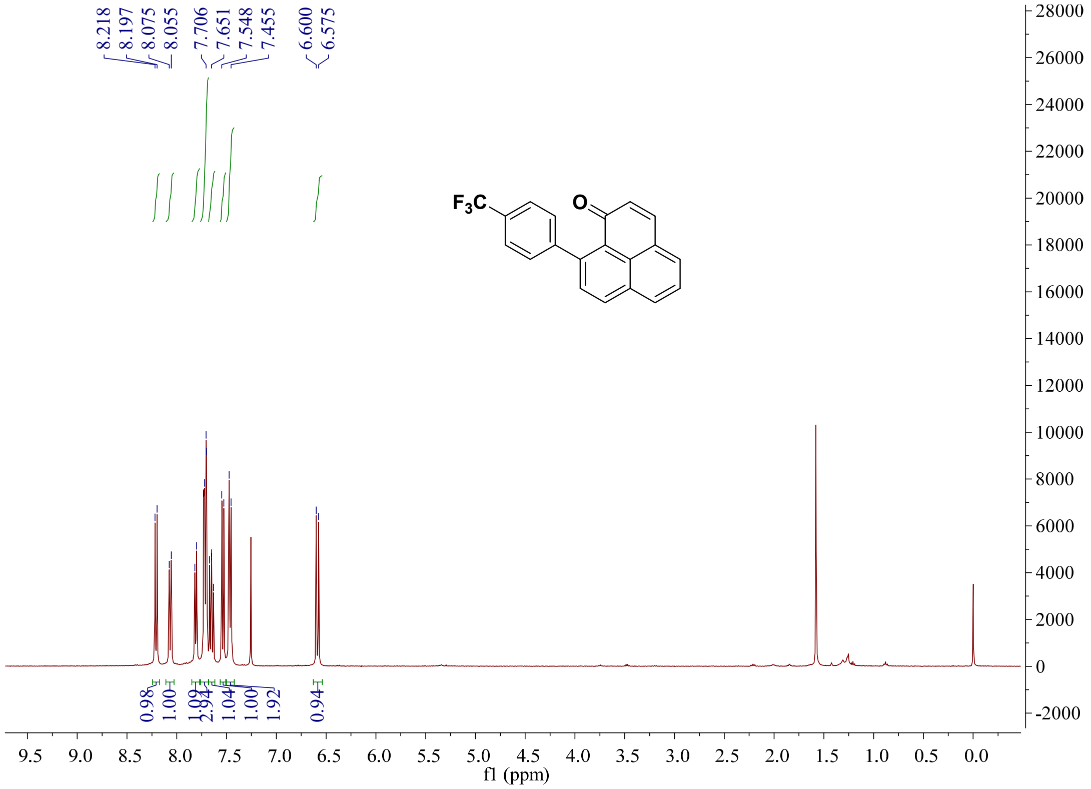

Figure S2. ^{1}H NMR (400 MHz, $CDCl_3$) spectra of PPN1

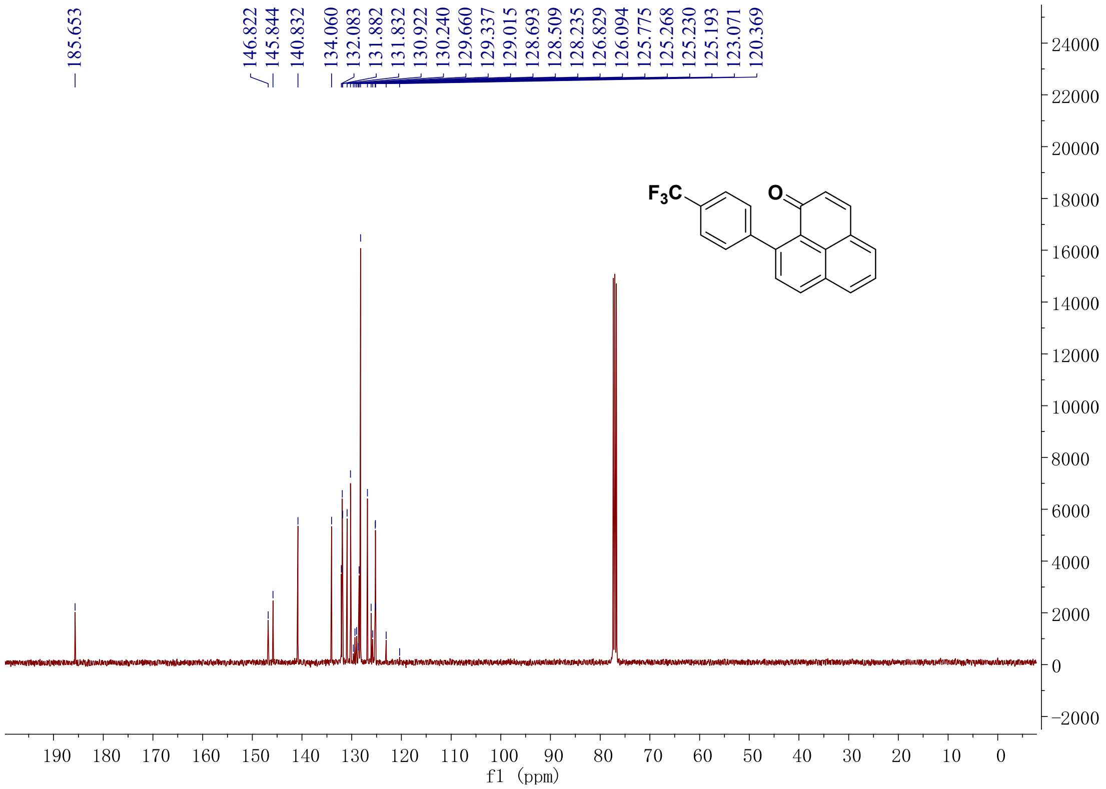

Figure S3. ^{13}C NMR (100 MHz, $CDCl_3$) spectra of PPN1

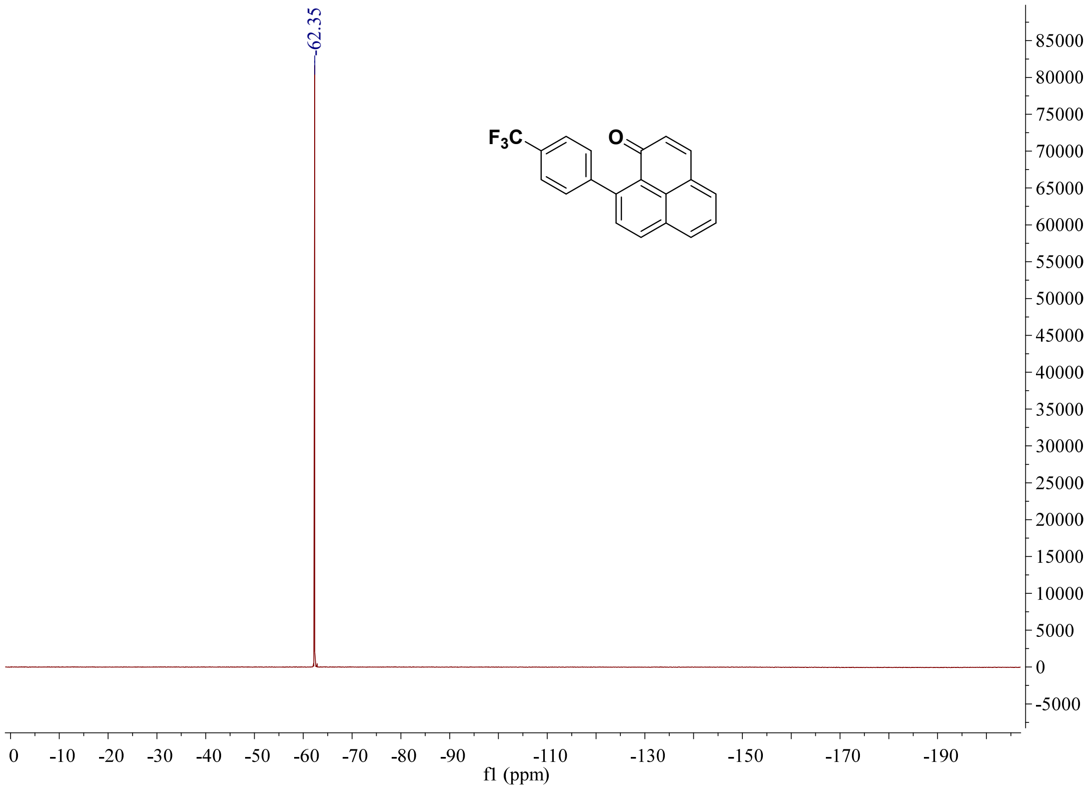

Figure S4. ^{19}F NMR (376 MHz, $CDCl_3$) spectra of PPN1

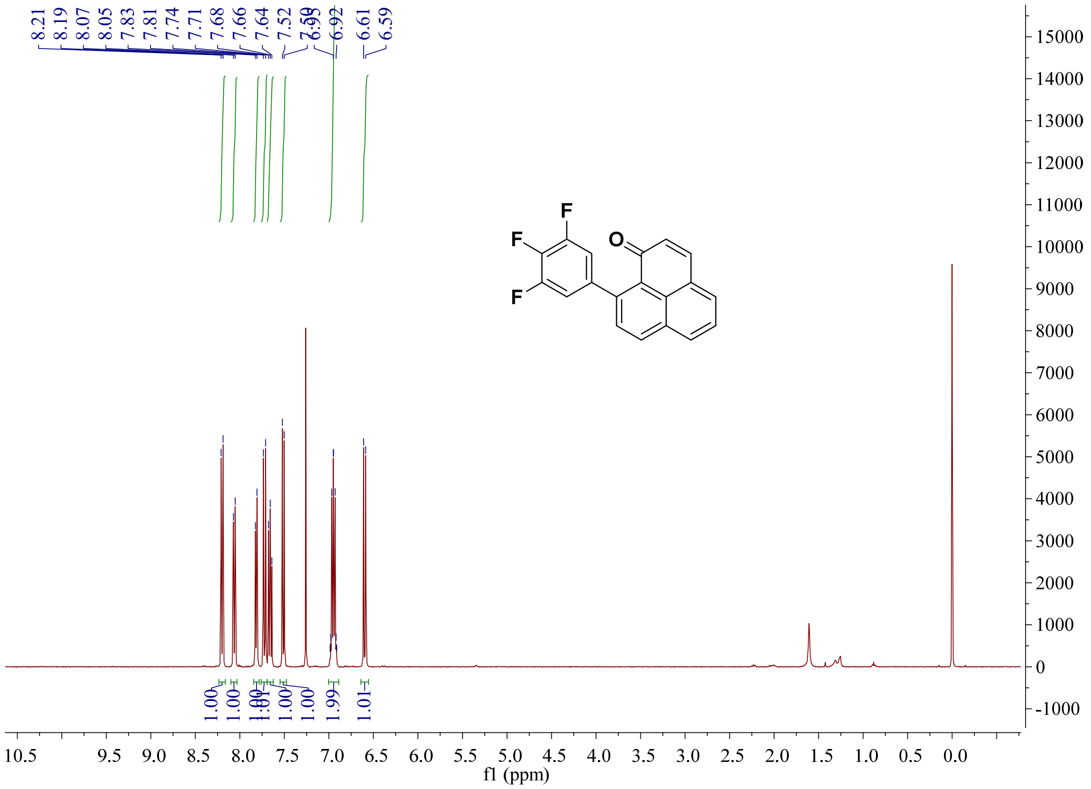

Figure S5. ^{1}H NMR (400 MHz, $CDCl_3$) spectra of PPN2

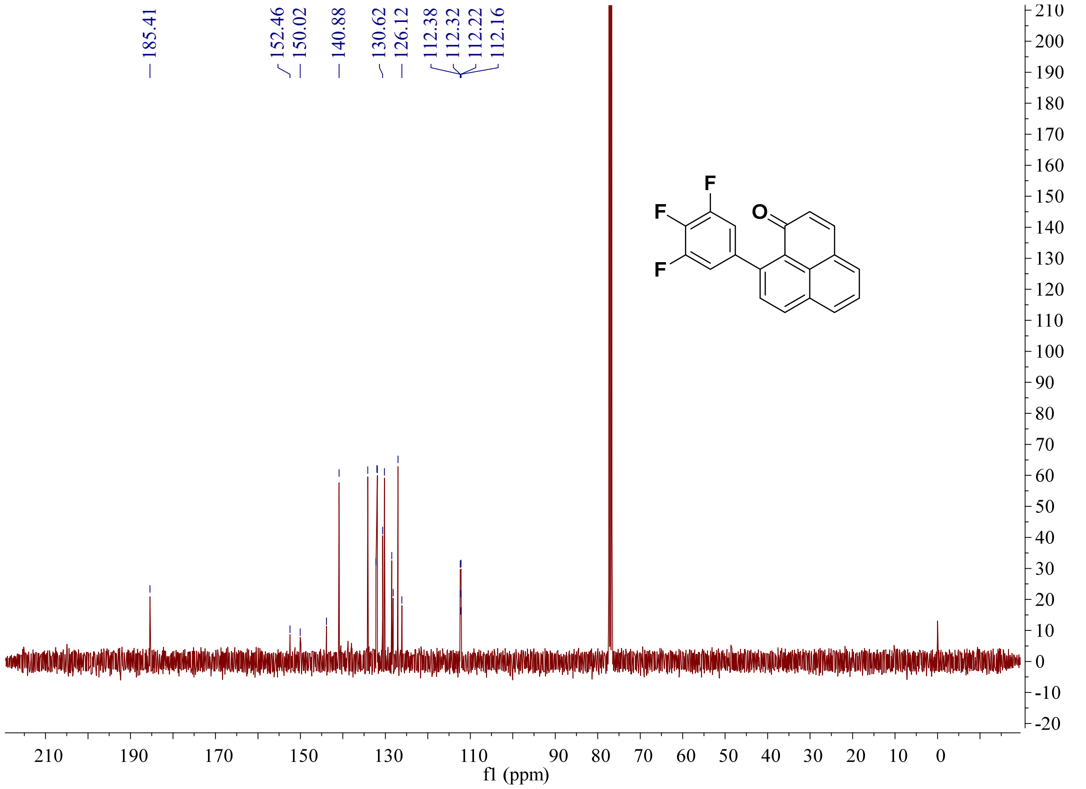

Figure S6. ^{13}C NMR (100 MHz, $CDCl_3$) spectra of PPN2

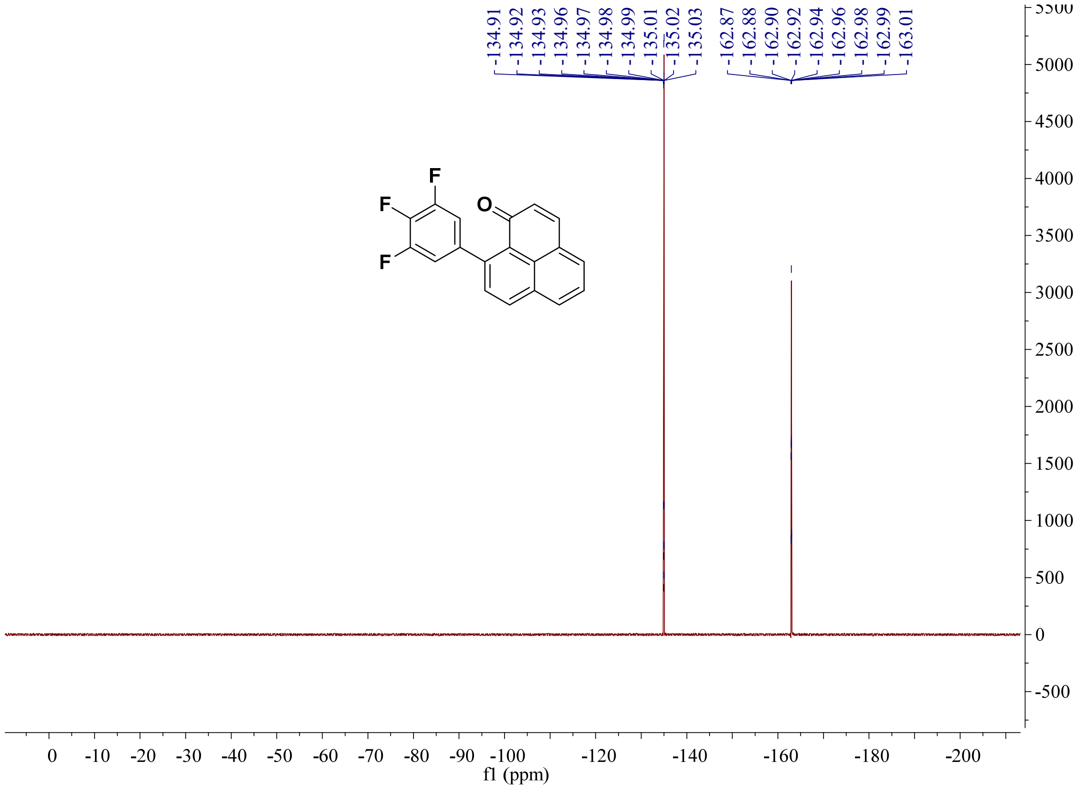

Figure S7. ^{19}F NMR (376 MHz, $CDCl_3$) spectra of PPN2

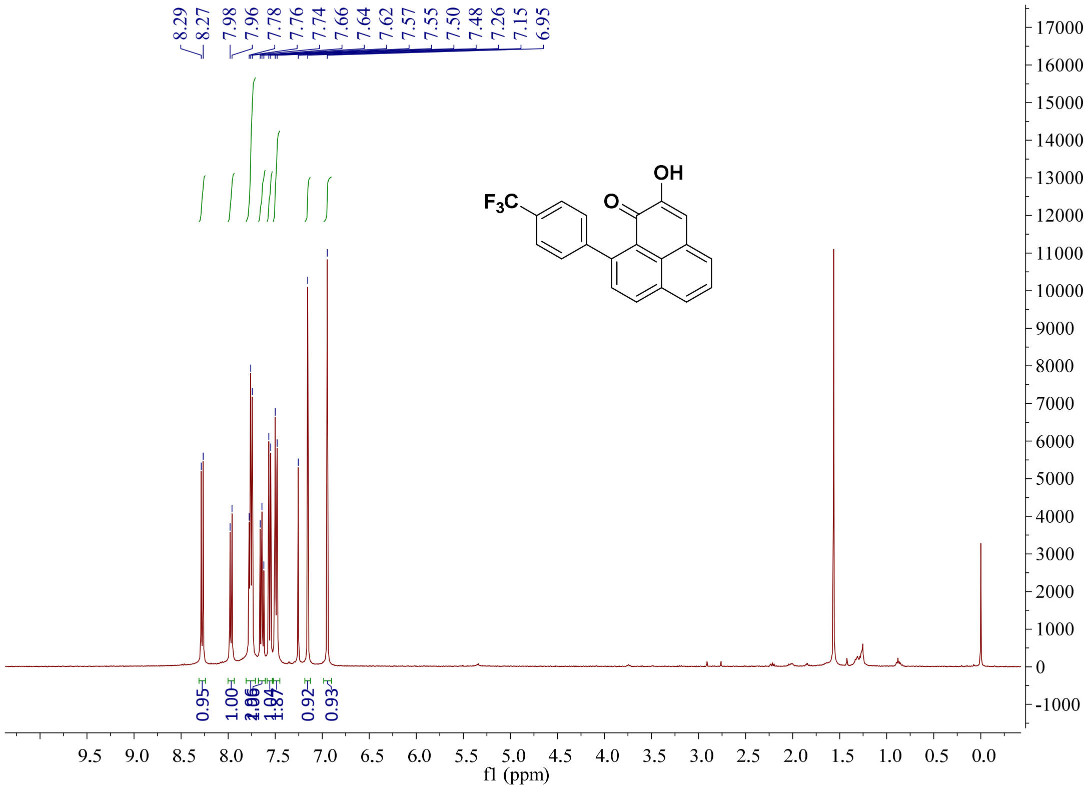

Figure S8. ^{1}H NMR (400 MHz, $CDCl_3$) spectra of HPPN1

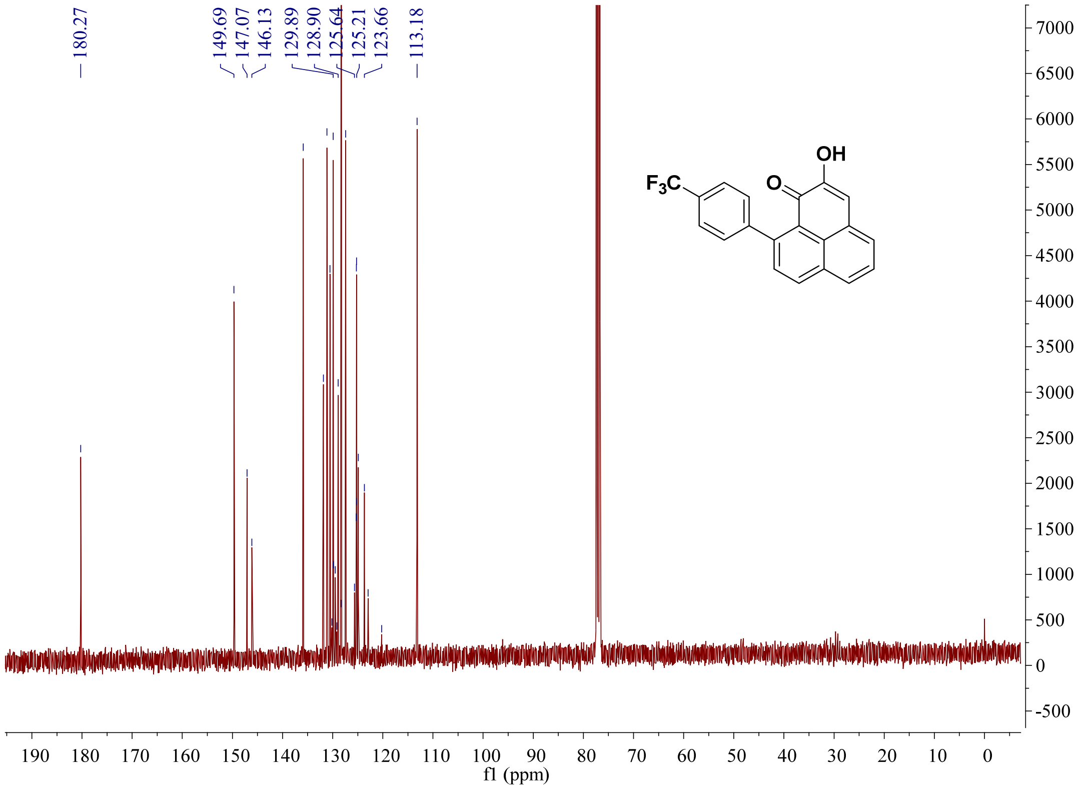

Figure S9. ^{13}C NMR (100 MHz, $CDCl_3$) spectra of HPPN1

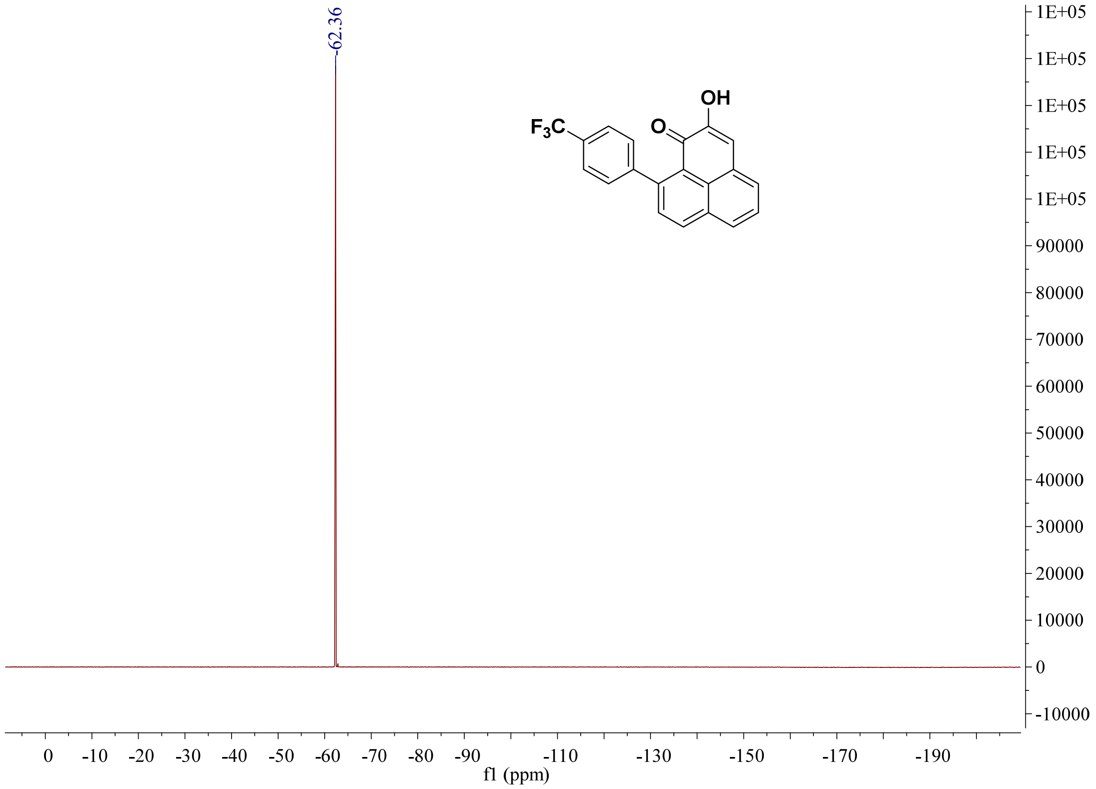

Figure S10. ^{19}F NMR (376 MHz, CDCl3) spectra of HPPN1

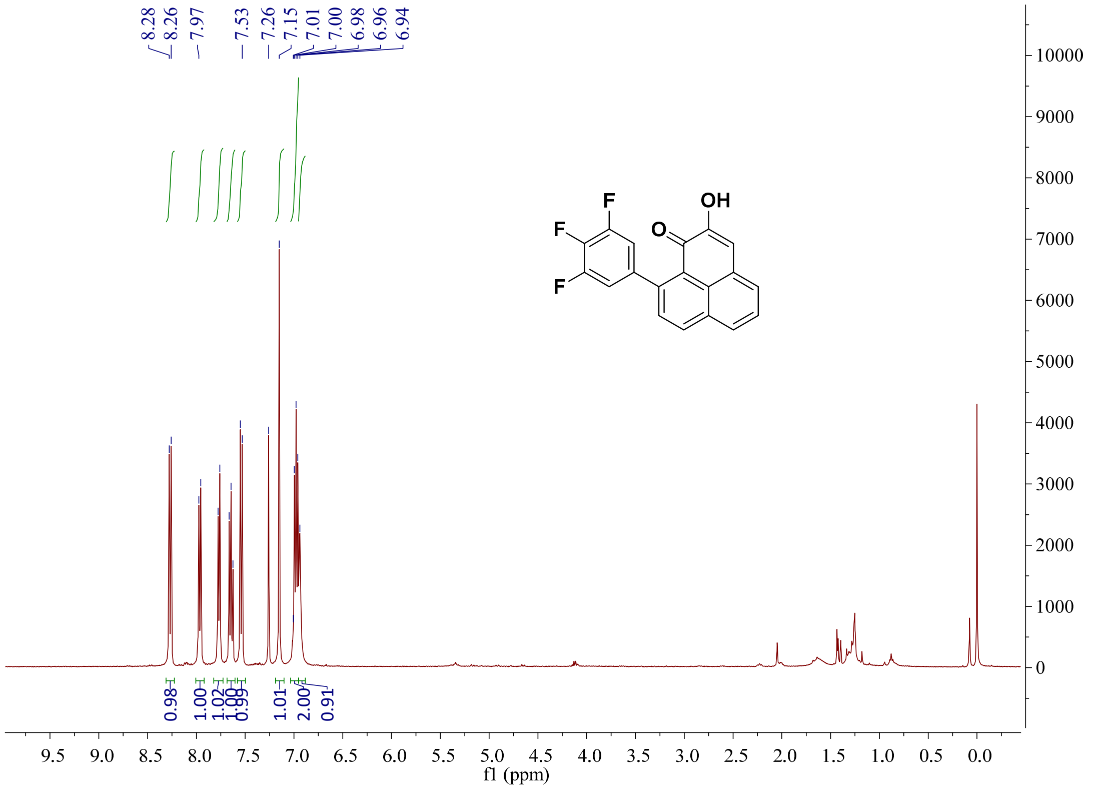

Figure S11. ^{1}H NMR (400 MHz, CDCl3) spectra of HPPN2

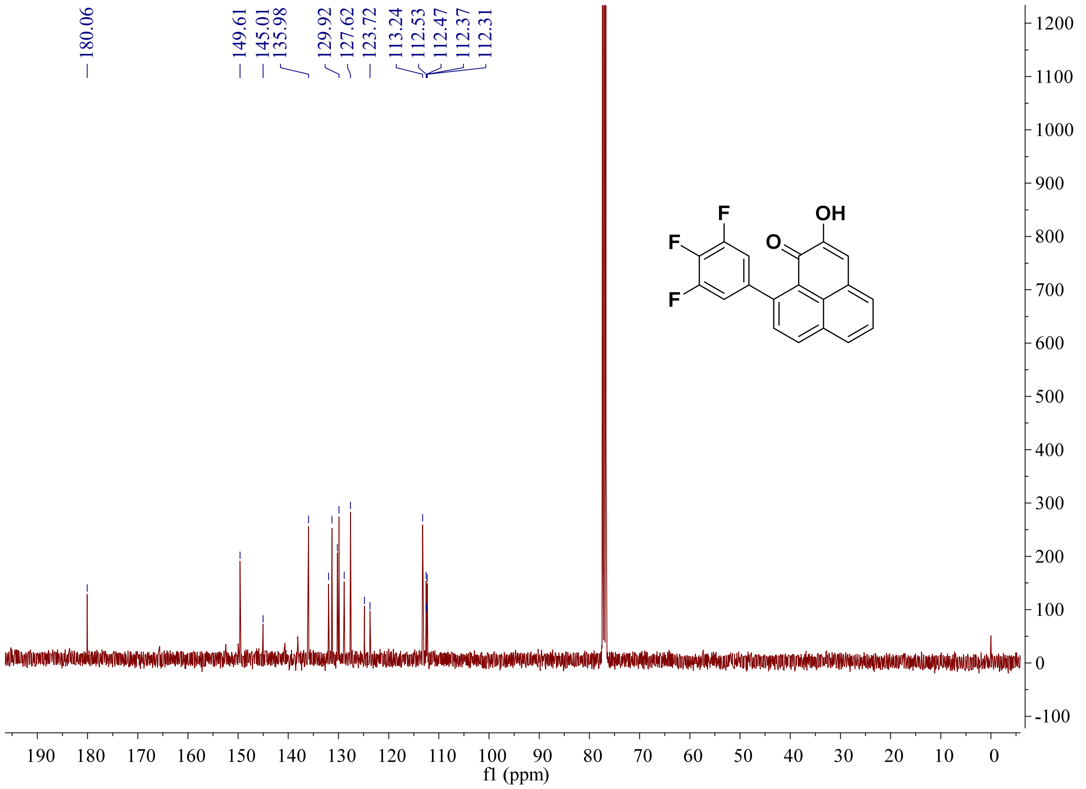

Figure S12. ^{13}C NMR (100 MHz, $CDCl_3$) spectra of HPPN2

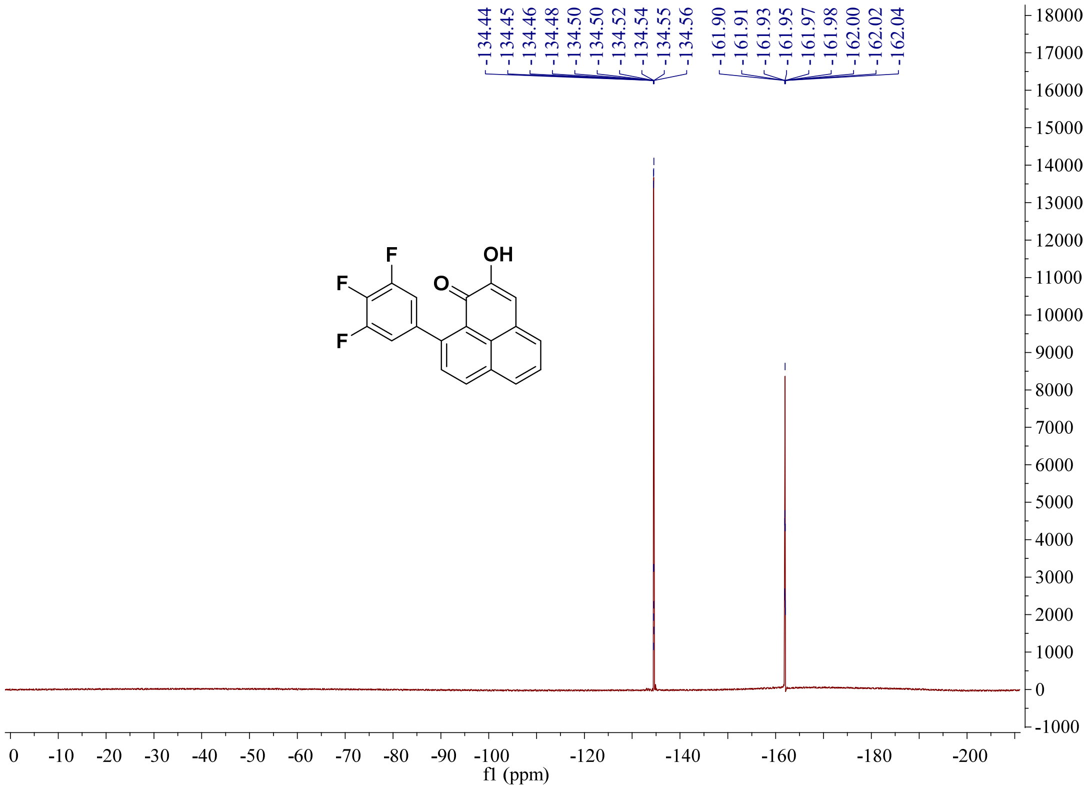

Figure S13. ^{19}F NMR (376 MHz, $CDCl_3$) spectra of HPPN2

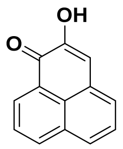

Figure S14. ^{1}H NMR (400 MHz, $CDCl_3$) spectra of HPPN3

Figure S15. ^{13}C NMR (100 MHz, $CDCl_3$) spectra of HPPN3

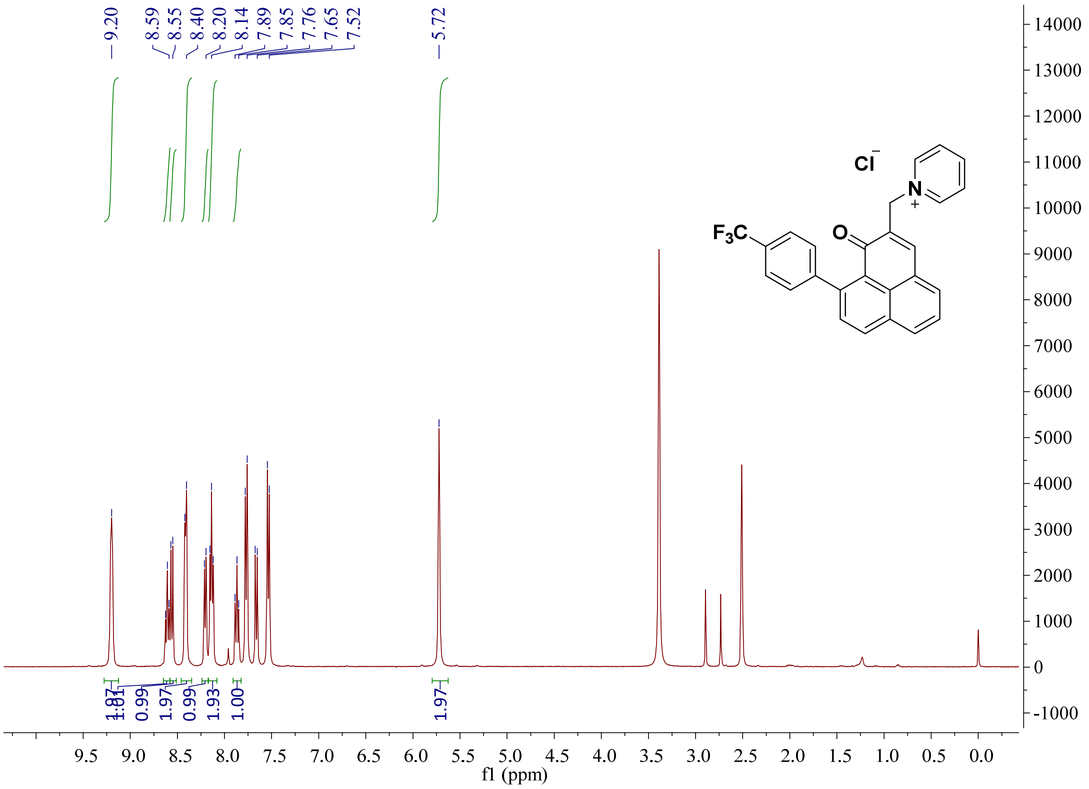

Figure S16. ^{1}H NMR (400 MHz, DMSO-d_6) spectra of PPPN1

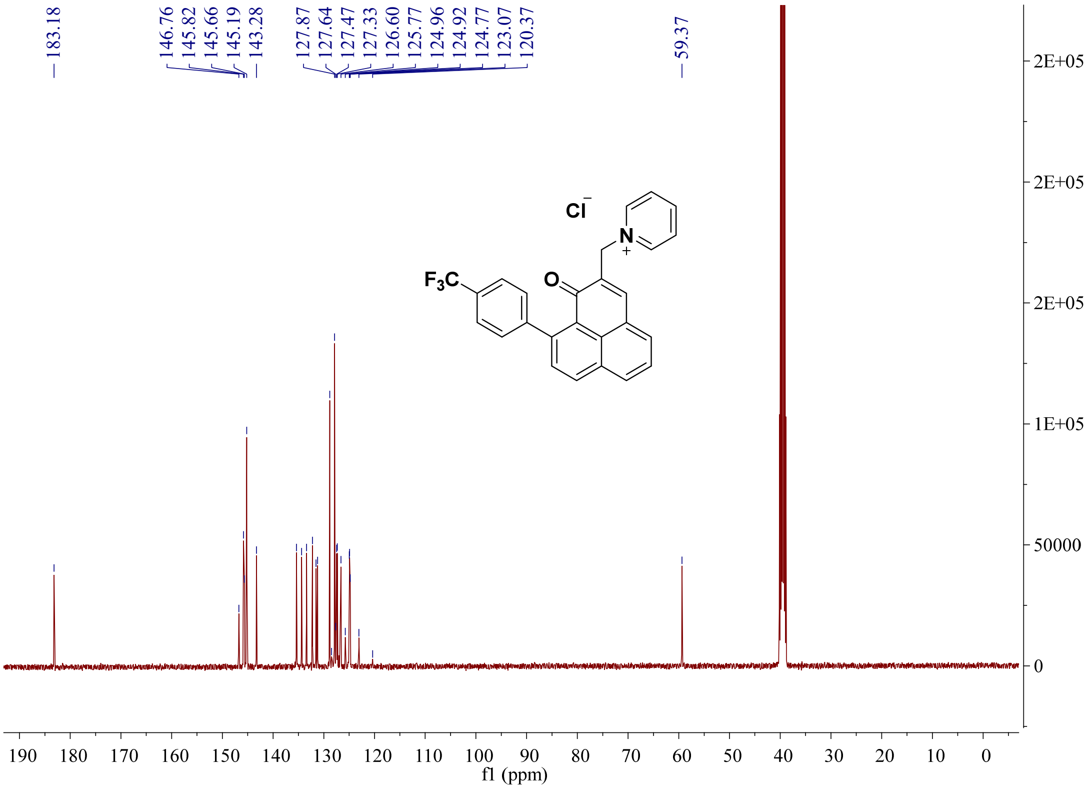

Figure S17. ^{13}C NMR (100 MHz, DMSO-d_6) spectra of PPPN1

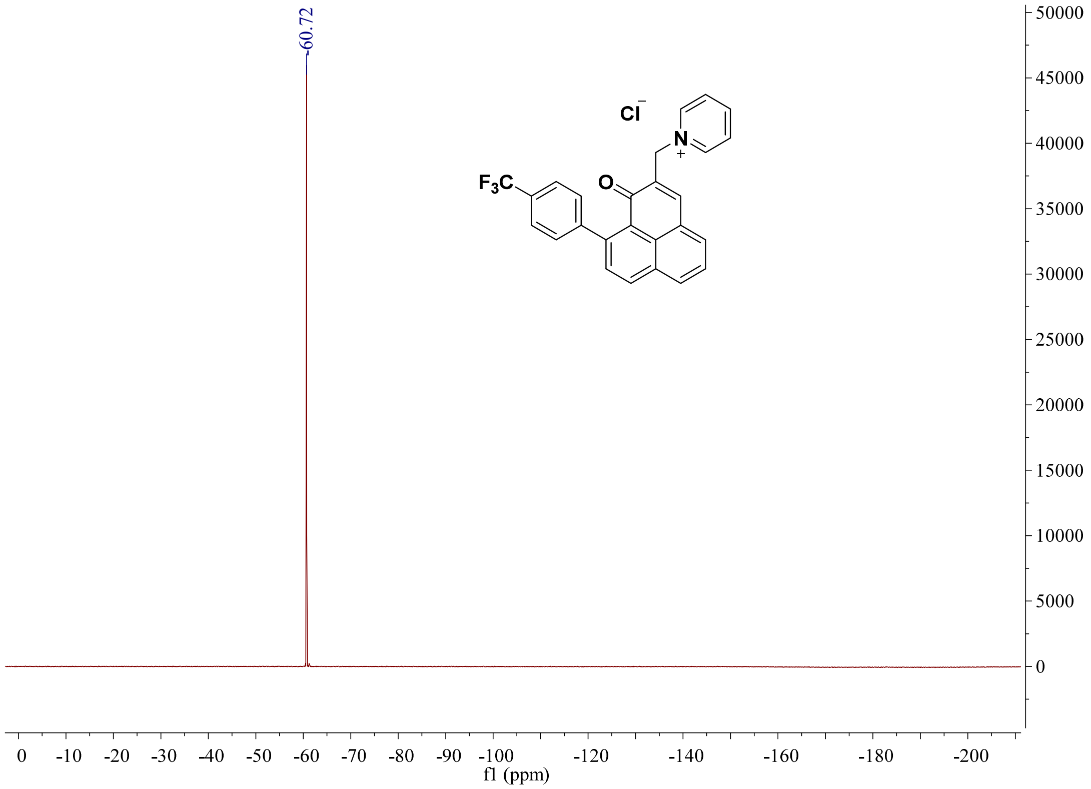

Figure S18. ^{19}F NMR (376 MHz, DMSO-*d6*) spectra of PPPN1

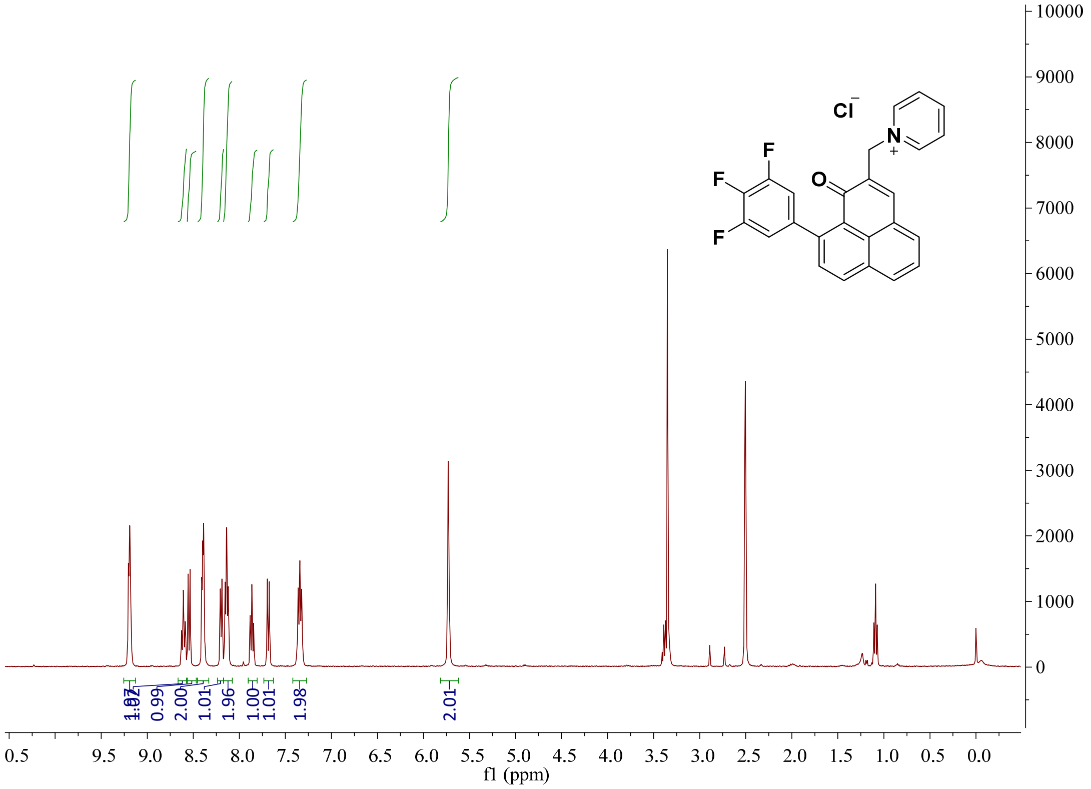

Figure S19. ^{1}H NMR (400 MHz, DMSO-*d6*) spectra of PPPN2

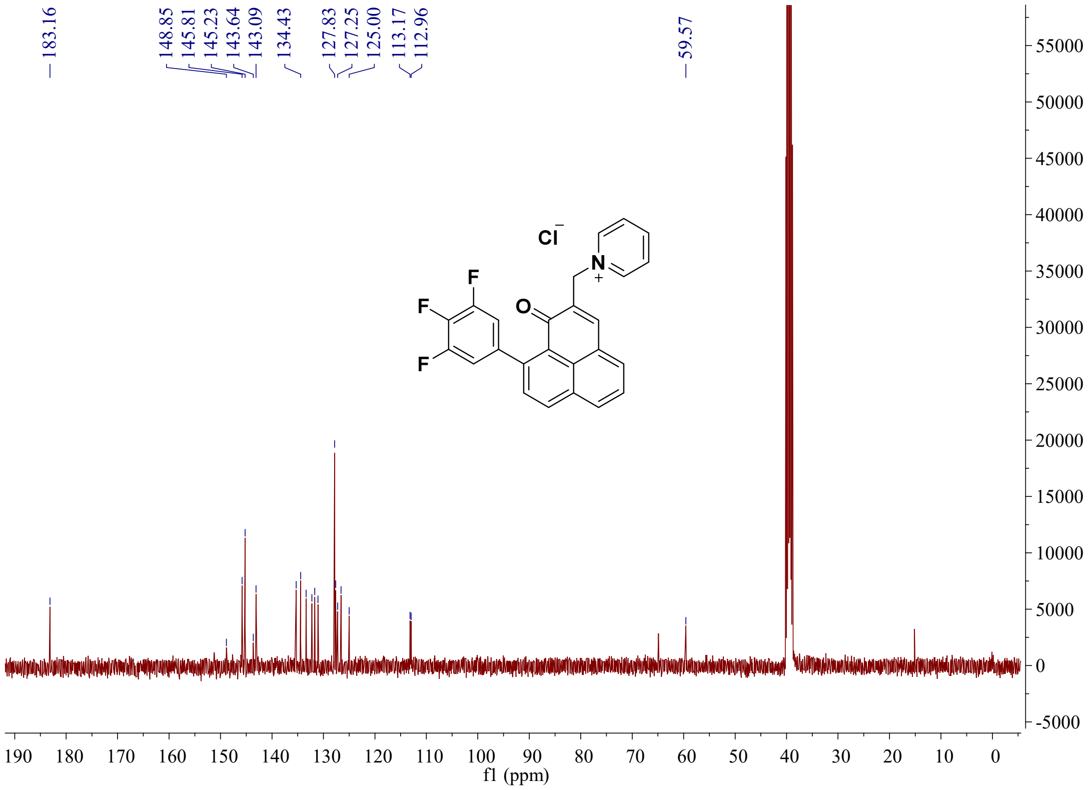

Figure S20. ^{13}C NMR (100 MHz, DMSO-d_6) spectra of PPPN2

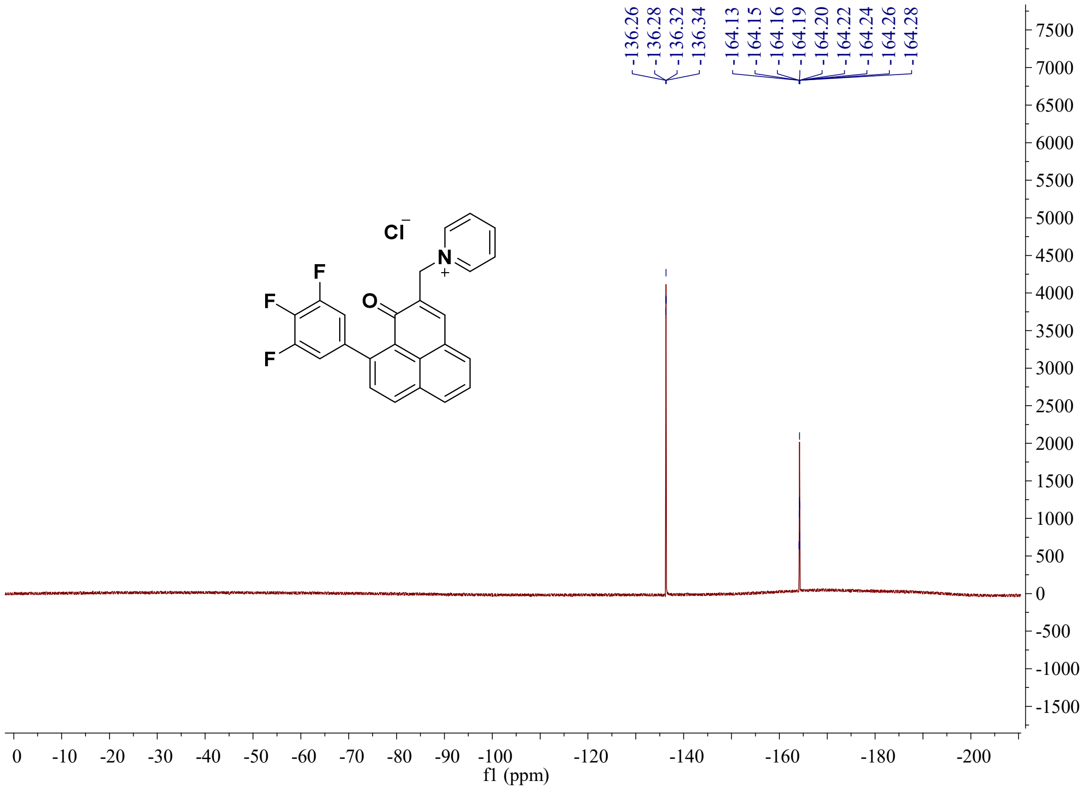

Figure S21. ^{19}F NMR (376 MHz, DMSO-d_6) spectra of PPPN2

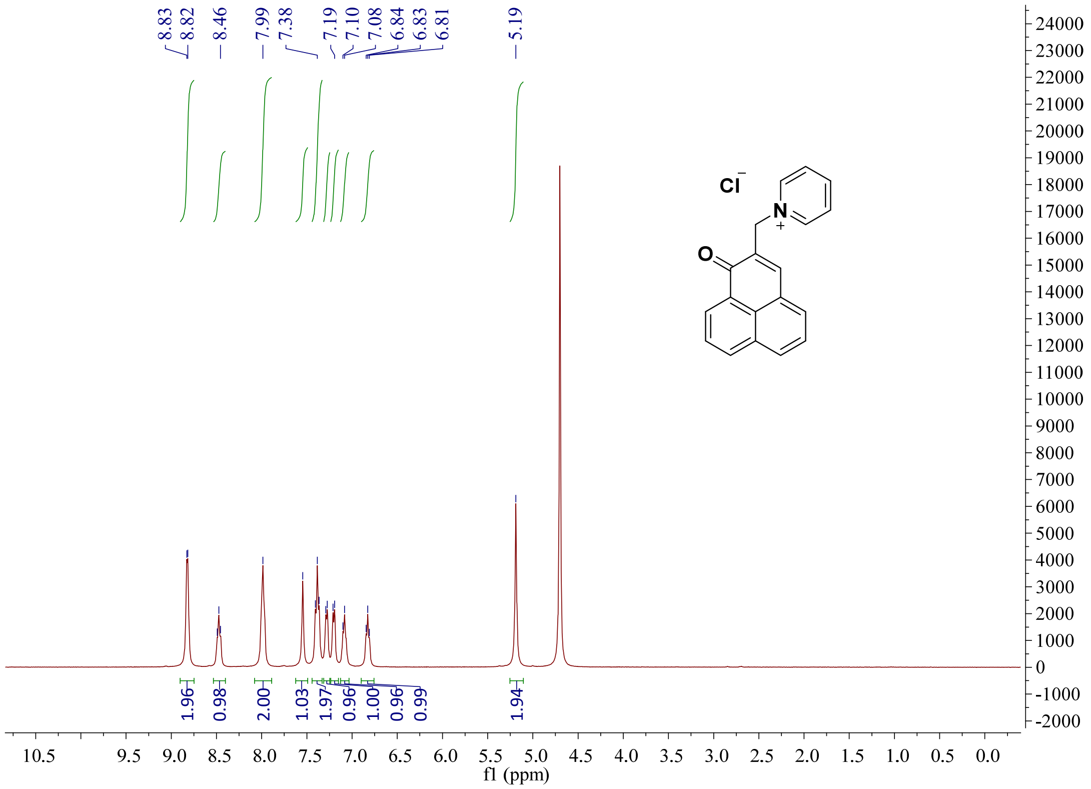

Figure S22. ^{1}H NMR (400 MHz, D_2O) spectra of PPPN3

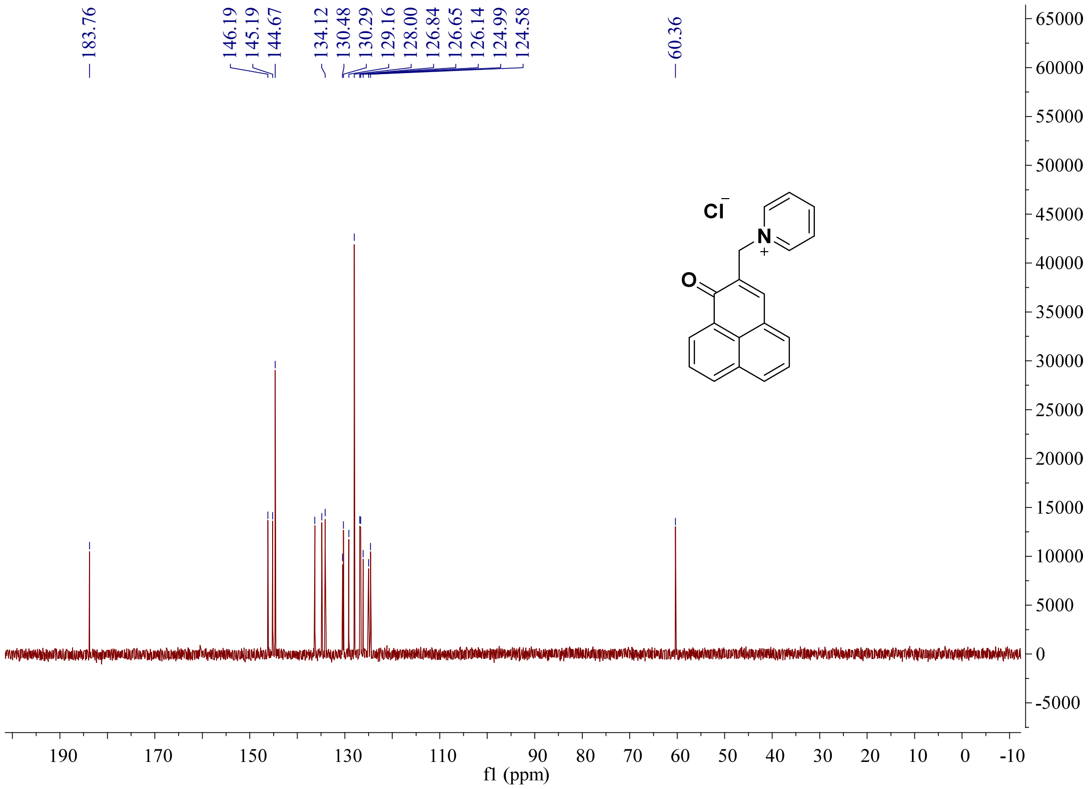

Figure S23. ^{13}C NMR (100 MHz, D_2O) spectra of PPPN3

5. Chemical oxidation detection of singlet oxygen

A chemical oxidation method based on ABDA was used to assess the capability of PN derivatives to generate $^{1}O_2$. A solution of PN derivative (3 mL in ultrapure water) containing 50 μL 9,10-anthracenediylbis (methylene) dimalonic acid (ABDA) solution (10 mmol/L in DMSO) was irradiated

with a blue light LED lamp (7 W). The control experiment was carried out with 3 mL ultrapure water containing 50 μL ABDA solution and the same irradiation but in the absence of PN derivative. The change in ABDA absorption at 400 nm was recorded as a function of irradiation time. Furthermore, the $^1O_2$ quantum yield of PN derivative was measured according to Equation S1, where Φ is the quantum yield of $^1O_2$ and K is the slope of the bleaching curve, R denotes reference, and S represents the sample.

$$\Phi_S = \Phi_R \frac{K_S}{K_R} \qquad \text{(Equation. S1)}$$

ABDA can react with $^1O_2$ quickly and produce a steady-state endoperoxide for UV detection. The reduction rates of absorbance intensity at 400 nm of ADBA in the presence of PN and PN derivatives are rapid. In contrast, the control experiment showed that pure ABDA without PN and PN derivatives was bleached to a small extent. By using compound PN as Φ_{PN} = 1.0, the $^1O_2$ quantum yields of PN and PN derivatives were calculated.

6. Photoactivated insecticidal activity

6.1 Photoactivated activity of *Aedes albopictus* larvae

Photoactivated insecticidal activity was determined as described previously.[5, 6] The test insects were the 4th-instar larvaes of *A. albopictus* (Skuse) which were obtained from National South pesticide initiative Center in Shanghai, China. PN was dissolved and serially diluted with acetone and compound **PPPN1**-**2** was dissolved and serially diluted with dechlorinated water. Each serial solution (0.2 mL) was added to a beaker containing 10 mL of dechlorinated water, and then 15 larvae were transferred into the dactylethrae. Two sets of experiments were performed for each compound, one of which was for light-treated trials, and another was cultivated in the dark throughout the trials. After 3 h incubation in dark, the light-treated groups were irradiated with blue light for 3 h, and then returned to darkness for 24-h incubation. The average mortality of three replications at each concentration was calculated, and the LC_{50} value was determined. All the experiments were conducted at least two times with three replicates in each case.
The pictures of *A. albopictus* larvae were taken by a polarizing microscope.

6.2 FITC uptake

Fluorescein isothiocyanate (FITC) uptake was analyzed by soaking *A. albopictus* larvaes in 10 mL of 100 mg/L Fluorescein isothiocyanate in M9 buffer (43.6 mM Na_2HPO_4, 22 mM KH_2PO_4, 2.1 mM NaCl, 4.7 mM NH_4Cl) with 100 mg/L PPPN1, 10 mg/L fipronil or without any other compound. Two sets of experiments were performed for each compound, one of which was for light-treated trials, and another was cultivated in the dark throughout the trials. After 24 h, the larvaes were washed three times with M9 buffer. FITC uptake was observed by fluorescence, using a fluorescence microscope with blue exciting light.

6.3 Photoactivated activity of root-knot nematode

M. incognita population Calissane were grown on *Lycopersicon esculentum* plants in a greenhouse, and collected as described by Rosso and associates.[7] Then hatched J2 were collected as described in Petri Plate Techniqueas [8].
PN and PPPN**1**-**2** was dissolved with DMF and serially diluted with dechlorinated water. Each serial

solution (0.05 mL) was added to three wells of 96-well platers and then 0.05 mL water containing root-knot nematode (J2) was transferred into the 96-well plate. Two sets of experiments were performed for each compound, one of which was for light-treated trials, and another was cultivated in the dark throughout the trials. After 3 h incubation in dark, the light-treated groups were irradiated with blue light for 1 h, 2 h, 3 h and 4 h, and then returned to darkness for 24 h incubation. The average mortality of three replications at each concentration was calculated. All the experiments were conducted at least two times with three replicates in each case. The pictures of *M. incognita* were taken by a polarizing microscope.

References

1. Li, Y.-Y.; Yan, B.; Qiao, X.-F., Visible light excitation and near-infrared luminescence of organo-lanthanide hybrids with mesoporous silica through 9-hydroxyphenalen-1-one linkage. *Microporous and Mesoporous Materials* **169**, 60-66 (2013).
2. Duque, L.; Zapata, C.; Rojano, B.; Schneider, B.; Otálvaro, F., Radical Scavenging Capacity of 2,4-Dihydroxy-9-phenyl-1H-phenalen-1-one: A Functional Group Exclusion Approach. *Organic Letters* **15**, 3542-3545 (2013).
3. Quinones, W.; Escobar, G.; Echeverri, F.; Torres, F.; Rosero, Y.; Arango, V.; Cardona, G.; Gallego, A., Synthesis and antifungal activity of Musa phytoalexins and structural analogs. *Molecules* **5**, 974-980 (2000).
4. Spath, A.; Leibl, C.; Cieplik, F.; Lehner, K.; Regensburger, J.; Hiller, K. A.; Baumler, W.; Schmalz, G.; Maisch, T., Improving photodynamic inactivation of bacteria in dentistry: highly effective and fast killing of oral key pathogens with novel tooth-colored type-II photosensitizers. *Journal of medicinal chemistry* **57**, 5157-5168 (2014).
5. Shao, G.; Jiang, D. X.; Xu, H. H.; Zeng, W.; Yu, H. J.; Tian, Y. Q., Synthesis and photoactivated insecticidal activity of tetraethynylsilanes. *J. Photochem. Photobiol. B.* **98**, 52-56 (2010).
6. Tian, Y.; Wei, X.; Xu, H., Photoactivated Insecticidal Thiophene Derivatives from Xanthopappus subacaulis. *Journal of Natural Products* **69**, 1241-1244 (2006).
7. Rosso, M.-N.; Favery, B.; Piotte, C.; Arthaud, L.; De Boer, J. M.; Hussey, R. S.; Bakker, J.; Baum, T. J.; Abad, P., Isolation of a cDNA Encoding a β-1,4-endoglucanase in the Root-Knot Nematode Meloidogyne incognita and Expression Analysis During Plant Parasitism. *Molecular Plant-Microbe Interactions* **12**, 585-591 (1999).
8. Ravichandra, N. G., Nematological Techniques. 300-301 (2014).
